# Precision‐Guided Stealth Missiles in Biomedicine: Biological Carrier‐Mediated Nanomedicine Hitchhiking Strategy

**DOI:** 10.1002/advs.202504672

**Published:** 2025-05-08

**Authors:** Yuyan Zhou, Xinyue Wang, Deyu Zhang, Hanxiao Cui, Xiaorong Tian, Wei Du, Zhenghui Yang, Dongling Wan, Zhiwei Qiu, Chao Liu, Zhicheng Yang, Lizhihong Zhang, Qiusheng Yang, Xuefeng Xu, Wenhao Li, Dong Wang, Haojie Huang, Wencheng Wu

**Affiliations:** ^1^ Central Laboratory and Department of Medical Ultrasound Sichuan Academy of Medical Sciences Sichuan Provincial People's Hospital University of Electronic Science and Technology of China Chengdu Sichuan Province 610072 China; ^2^ Department of Gastroenterology Shanghai Institute of Pancreatic Diseases Changhai Hospital National Key Laboratory of Immunity and Inflammation Naval Medical University Shanghai 200433 China; ^3^ Department of Stomatology Zhuhai Campus of Zunyi Medical University Zhuhai Guangdong Province 519041 China; ^4^ Department of Infectious Diseases Fujian Provincial Hospital Fuzhou Fujian 350001 China; ^5^ Department of Gastroenterology Fujian Provincial Hospital Fuzhou Fujian 350001 China

**Keywords:** biological carrier‐mediated nanomedicine hitchhiking strategy (BCM‐NHS), covalent conjugation, encapsulation, ligand–receptor interactions, non‐covalent conjugation

## Abstract

Nanodrug delivery systems (NDDS) have demonstrated broad application prospects in disease treatment, prevention, and diagnosis due to several advantages, including functionalization capability, high drug‐loading capacity, drug stability protection, and the enhanced permeability and retention (EPR) effect. However, their clinical translation still faces multiple challenges, including rapid clearance by the reticuloendothelial system (RES), poor targeting specificity, and insufficient efficiency in crossing biological barriers. To address these limitations, researchers have developed the biological carrier‐mediated nanomedicine hitchhiking strategy (BCM‐NHS), which leverages circulating cells, proteins, or bacteria as natural “mobile carriers” to enhance drug delivery. This approach enables nanocarriers to inherit the intrinsic biological properties, endowing them with immune evasion, prolonged circulation, dynamic targeting, biocompatibility, biodegradability, and naturally optimized biological interfaces. Here, a systematic overview of the BCM‐NHS is provided. First, the review delves into the methods of nanoparticles (NPs) binding and immobilization, encompassing both the surface‐attachment‐mediated “backpack” strategy and the encapsulation‐based “Trojan horse” strategy. Second, the classification of biological carriers, including both cell‐based and non‐cell‐based carriers, is elucidated. Third, the physical properties and release mechanisms of these nanomaterials are thoroughly described. Finally, the latest applications of BCM‐NHS in therapeutic and diagnostic contexts across various disease models including tumor, ischemic stroke, and pneumonia are highlighted.

## Graphical abstract

Diagram illustrating the binding methods of NPs and biological barriers. The biological carrier‐mediated nanomedicine hitchhiking strategy (BCM‐NHS) utilizes two distinct techniques: the surface‐based “Backpack” method, which relies on ligand‐receptor binding, covalent conjugation, and non‐covalent binding, and the encapsulated “Trojan horse” approach, which employs neutrophils and monocytes/macrophages as natural delivery vehicles. Created with Biorender (Agreement number: GW2854RTK7).

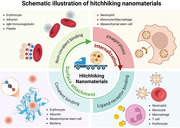

## 1 Introduction

Conventional systemic drug administration faces challenges in meeting the demands of precision medicine due to issues such as widespread drug distribution, insufficient drug concentration at disease sites, and significant toxicity. Engineered nanoparticles‐based drug delivery systems (NDDS) offer a revolutionary solution to these limitations,^[^ ^1^, ^2^ ^]^ with the following key advantages^[^ ^3^, ^4^, ^5^, ^6^, ^7^, ^8^ ^]^: 1) Functionalization capability and high drug loading capacity: nanoparticles (NPs) exhibit high drug‐loading capacity and versatile functionalization potential owing to their small size and large surface‐to‐volume ratio. 2) Drug stability protection: NPs shield therapeutic agents from enzyme‐induced degradation, prolong circulation time, and optimize biodistribution, thereby enhancing drug stability and solubility while reducing systemic toxicity.^[^ ^9^, ^10^, ^11^, ^12^ ^]^ 3) Enhanced permeability and retention (EPR) effect: leveraging their size advantage (5–250 nm), NPs gradually accumulate at tumor sites through the EPR effect.

Current NDDS still face challenges posed by four major physiological barriers,^[^ ^13^ ^]^ as detailed below: 1) Blood transport barrier: firstly, hemodynamic shear forces trigger structural damage to carriers, leading to premature drug release. Additionally, NPs are prone to rapid surface adsorption of plasma proteins, which accelerates their clearance by RES.^[^ ^14^ ^]^ Lastly, NPs smaller than 5.5 nm may undergo renal filtration from the bloodstream and subsequent urinary excretion.^[^ ^9^, ^10^, ^15^ ^]^ 2) Endothelial penetration barrier: Conventional NPs exhibit limited trans‐endothelial transport efficiency, struggling to permeate biological barriers such as the blood‐brain barrier.^[^ ^16^, ^17^, ^18^ ^]^ 3) Interstitial diffusion barrier: In complex physiological environments like tumor tissues, the interstitial space exists in a hypertensive state with dense extracellular matrix (ECM), resulting in low penetration efficiency of nanomedicines and poor infiltration into deep lesion regions to exert therapeutic effects.^[^ ^19^ ^]^

**Figure 1:**
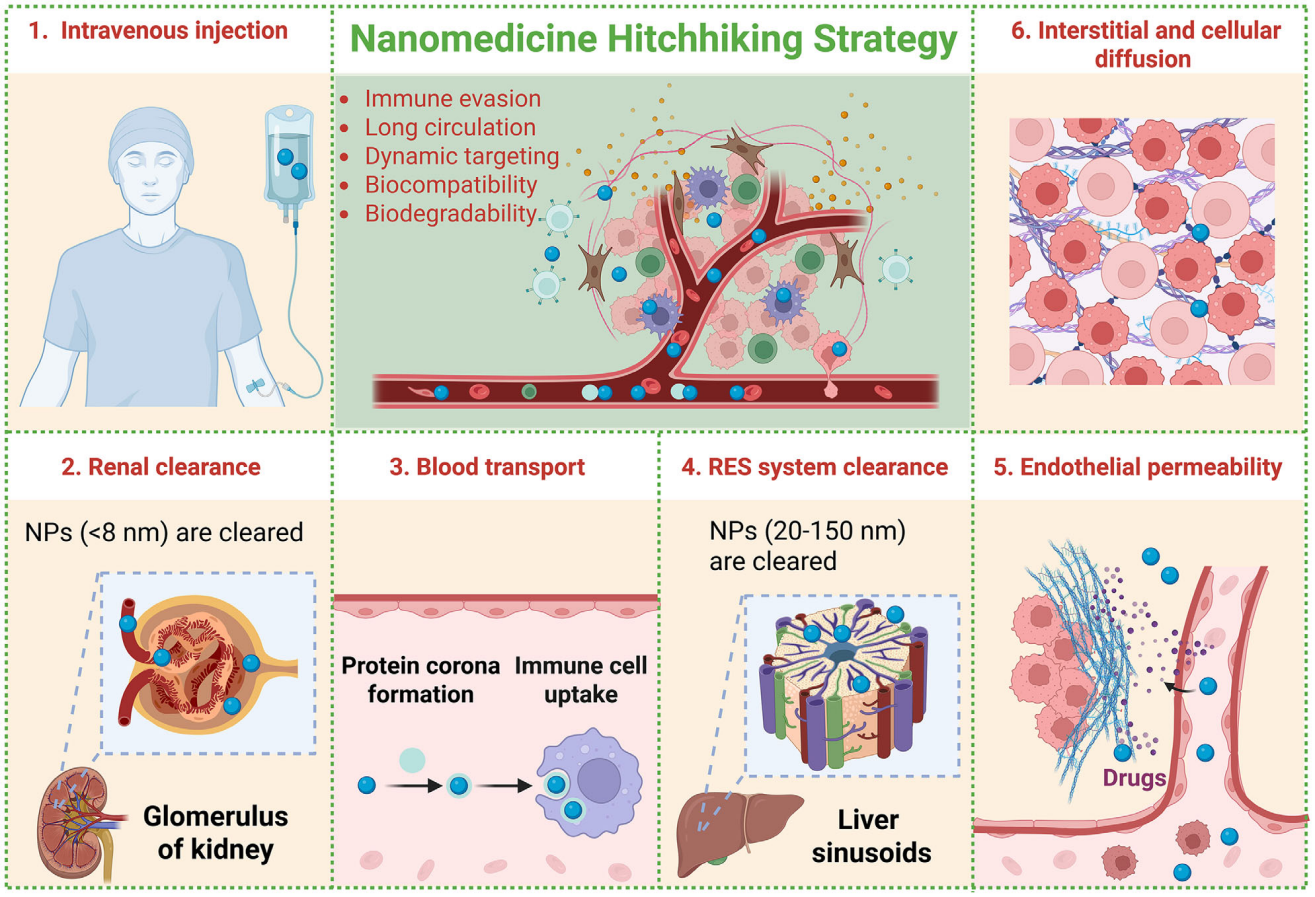
Diagram illustrating the in vivo dynamics of NPs and biological barriers: 1) NPs enter blood via intravenous injection; 2) NPs (<8 nm) undergo renal clearance by glomerular filtration; 3) NPs form a “protein corona” in blood, risking immune uptake; 4) NPs (20–150 nm) are cleared by Kupffer macrophages in liver sinusoids; 5) NPs require vascular margination and endothelial penetration to reach tissues; 6) Some NPs cross cell membranes for intracellular delivery. BCM‐NHS overcomes these barriers through immune evasion, prolonged circulation, dynamic targeting, biocompatibility, and biodegradability. Created with Biorender (Agreement number: HP2853V5SK).

The biological carrier‐mediated nanomedicine hitchhiking strategy (BCM‐NHS) refers to leveraging the natural tropism or transport properties of biological carriers (e.g., cells, proteins, or molecules) to deliver drugs or therapeutic agents to specific target sites.^[^ ^20^, ^21^ ^]^ For example, treating acute kidney injury (AKI) with traditional nanomedicines is challenging due to their low reactive oxygen species (ROS) scavenging efficacy and poor inflammatory chemotaxis. To address this, a novel cerium single‐atom catalyst (A‐CeSACs) has been developed. This catalyst hitches a ride on neutrophils to enter the AKI inflammatory environment and has demonstrated remarkably enhanced catalytic therapeutic efficacy in glycerol‐induced AKI mouse models.^[^ ^22^ ^]^ The advantages of BCM‐NHS are as follows^[^ ^23^, ^24^, ^25^, ^26^, ^27^ ^]^: 1) Immune evasion and prolonged circulation: Unlike synthetic nanocarriers that often trigger immune responses, endogenous biological carriers (e.g., RBCs, platelets, leukocytes) inherently express “self‐marker” proteins (e.g., CD47) and possess surface compositions that evade phagocytic clearance. This intrinsic stealth capability significantly extends their circulation half‐life, ensuring sustained drug delivery; 2) Dynamic and autonomous targeting: Traditional active targeting strategies rely on pre‐conjugated ligands, which are often limited by heterogeneous target expression and physiological barriers (e.g., endothelial tight junctions). In contrast, hitchhiked nanomaterials leverage the natural tropism of their biological carriers—such as platelets' inflammation‐directed migration or leukocytes' tumor‐homing ability—to dynamically localize at disease sites. This physiological signal‐guided targeting enables traversal of otherwise impermeable biological barriers (e.g., the blood‐brain barrier or fibrotic tumor stroma) for precise lesion accumulation; 3) Intrinsic biocompatibility and biodegradability: Synthetic carriers frequently face challenges related to polymer toxicity, non‐degradable byproducts, or unpredictable pharmacokinetics. BCM‐NHS circumvents these issues, as the host‐derived vehicles are naturally biocompatible and undergo physiological degradation pathways, minimizing off‐target toxicity and long‐term accumulation risks. 4) Naturally optimized biological interfaces: While conventional nanocarriers require complex surface modifications for functionality, BCM‐NHS often simplify production by utilizing naturally optimized biological interfaces. For example, platelet membranes inherently preserve adhesion molecules (e.g., P‐selectin) for inflammatory targeting without additional engineering (**Figure** **1**).

Therefore, this article provides a systematic overview of the BCM‐NHS, structured as follows: Initially, we delve into the methods of NPs binding and immobilization, encompassing both the surface‐attachment‐mediated “backpack” strategy and the encapsulation‐based “Trojan horse” strategy. Subsequently, we elucidate the classification of biological carriers, which includes both cell‐based and non‐cell‐based carriers. Following that, the physical properties and release mechanisms of these nanomaterials are thoroughly described. Lastly, we highlight the latest applications of BCM‐NHS in therapeutic and diagnostic contexts across various disease models.

## 2 Overview of Binding Methodologies

The BCM‐NHS utilizes living cells as carriers for drug delivery, classified into “ex vivo hitchhiking strategy” and “in situ hitchhiking strategy” based on compositional environments. Ex vivo hitchhiking strategy involves in vitro preparation of drug‐loaded cells followed by reinfusion into the body, offering controlled drug‐loading ratios but requiring labor‐intensive processes (carrier harvesting, quality control) and posing risks such as poor ex vivo stability, limited storage longevity, and potential inter‐individual immune rejection. In contrast, in situ hitchhiking strategy employs drug carriers with cell‐targeting specificity injected directly into the body; these carriers attach to or are internalized by target cells, migrating with them to accumulate at pathological sites,^[^ ^28^ ^]^ with advantages including simplified procedures, cost efficiency, extended drug stability, and reduced immunogenicity risks from cell transplantation. Classification by binding mechanisms yields “backpack” and “Trojan horse” approaches. The former immobilizes NPs on carrier cell surfaces via physical/chemical interactions utilizing ligand‐receptor binding, covalent conjugation, or non‐covalent adsorption^[^ ^13^ ^]^ (**Table** **1**).

**Table 1:** The advantages and disadvantages of different attachment methods.

Attachment Strategy	Attachment method	Advantages	Disadvantages	Ideal carrier types	Refs.
Surface‐attachment‐mediated “backpack” strategy	Non‐covalent	1) No complex modifications required for cells or nanoparticles. 2) Simple operation, suitable for nanoparticles with positive charge or sufficient hydrophobicity.	1) The binding force may not be strong, and nanoparticles may unpredictably detach in the body. 2) Positively charged nanoparticles may have potential toxicity to cells.	Red blood cells, albumin, immunoglobulins, platelets, etc.	[29, 30, 31, 32, 33, 34, 35, 36, 37, 38, 39]
	Ligand‐receptor attachment	1) Reliable and reproducible attachment through naturally existing ligand‐receptor interactions without cell modification. 2) Can design attachment platforms for various cell types, suitable for large‐scale production.	1) If the binding affinity between ligand and receptor is not strong enough, it may not be maintained until the target site. 2) May lead to attachment to non‐target cells or trigger specific biological responses.	Neutrophils, monocytes/macrophages, T cells, red blood cells, T cells, etc.	[40, 41, 42, 43, 44, 45, 46, 47, 48, 49, 50]
	Covalent coupling	1) Provides stronger covalent bonds than non‐covalent or ligand‐induced binding. 2) Avoids cell functions that may be triggered by receptor activation.	When the carrier is a circulating cell, permanent modification of the cell surface is required, which may affect the function of the carrier cell.	Albumin, red blood cells, etc.	[51, 52, 53, 54, 55, 56]
Encapsulation‐based “Trojan horse” strategy	Encapsulation	Does not alter the cell membrane, potentially protecting nanoparticles from interacting with non‐target tissues in the body.	After phagocytosis, degradable nanoparticles may degrade within the cell, leading to premature drug release.	Neutrophils, monocytes/macrophages, mesenchymal stem cells, etc.	[45, 57, 58, 59, 60, 61, 62, 63, 64, 65, 66, 67, 68, 69, 70, 71, 72]

The diverse hitchhiking strategies exhibit distinct advantages and limitations: non‐covalent methods require minimal modification of cells/NPs with operational simplicity but may suffer from weak adhesion and cytotoxicity from positively charged particles; ligand‐receptor binding leverages intrinsic receptor‐mediated interactions without cellular engineering, ensuring reliable attachment, though insufficient binding affinity could compromise targeting fidelity; covalent conjugation provides robust covalent linkages that circumvent receptor activation‐triggered cellular functions yet necessitates permanent surface modifications; encapsulation strategies preserve membrane integrity to shield NPs from non‐target interactions but risk intracellular degradation; additionally, leukocyte sensitivity to stimulation may alter their phenotype/function upon intracellular payload integration, potentially hindering immune therapy.^[^ ^73^ ^]^ Ultimately, optimal attachment methodology selection depends on target applications, specific cell types, and NPs properties, ideally ensuring secure NPs retention until destination‐site arrival.^[^ ^74^ ^]^

### 2.1 Surface Attachment‐Mediated “Backpack” Strategy

#### 2.1.1 “Backpack” Strategy by Ligand–Receptor Interactions

Ligand‐receptor interactions represent specific binding processes between biomolecules mediated by the precise “key‐lock” mechanism.^[^ ^75^, ^76^ ^]^ Leveraging this principle, the BCM‐NHS employs engineered surface modifications of nanocarriers to enable their precise anchoring onto endogenous carrier surfaces, thereby overcoming biological barriers inherent to conventional delivery systems and achieving efficient targeted delivery of nanomedicines to pathological sites such as tumors, inflammatory diseases, and cerebrovascular disorders. Current technological approaches primarily include: 1) Ligand modification strategies involving E‐selectin, sialic acid (SA), Ac‐PGP peptide, CFLFLF peptide, lipoteichoic acid (LTA), and monocyte chemoattractant protein 1 (MCP1); (2) Antibody conjugation strategies such as leukocyte/erythrocyte antibodies. Both approaches enhance delivery specificity by improving the molecular recognition accuracy of ligand‐receptor pairs.

The ligand‐receptor interaction‐mediated “backpack” strategy is widely applied to leukocytes, including neutrophils and monocytes/macrophages (**Table** **2**). Leveraging sialylated glycoconjugates on leukocyte surfaces that specifically bind E‐selectin, E‐selectin‐modified gemcitabine/PD‐1 inhibitor‐loaded liposomal NPs (ES‐GBL) anchor onto leukocytes, enabling targeted delivery to hepatocellular carcinoma regions to enhance immune response and suppress immune evasion.^[^ ^40^ ^]^ Utilizing C–C motif chemokine receptor 2 (CCR2) receptors on monocytes/macrophages that recognize MCP1, MCP1‐conjugated gadolinium micelles (MCP1‐Gd micelles) are transported by monocytes to metastatic lymph nodes, where gadolinium accumulation enhances MRI detection precision.^[^ ^41^ ^]^ Exploiting glucose transporter (GLUT)‐mediated chitosan binding on erythrocytes, surface‐modified chitosan NPs (SHIDS) achieve erythrocyte anchoring, dynamically regulating insulin release via glucose oxidase (GOx)‐catalyzed processes to mimic pancreatic β‐cell secretion kinetics.^[^ ^42^ ^]^ Capitalizing on PD‐1 antigen expression on T cells, PD‐1 antibody‐functionalized hemoglobin/catalase‐loaded metal‐organic framework/liposome hybrids (C&H@MOF/PL‐A) bind T cell surfaces in circulation. Upon tumor infiltration, these hybrids release hemoglobin (Hb) and catalase (CAT) to alleviate hypoxia and activate CD8^+^ T cell immunity, demonstrating therapeutic efficacy against colorectal cancer.^[^ ^43^ ^]^

**Table 2:** “Backpack” strategy by ligand‐receptor interactions.

Hitchhiking pathway	No.	Nanomedicine name	Nanomaterial structure	Hitchhiking cell	Ligand on nanomedicine	Receptor on hitchhiking cell	Loaded drug name	Role of loaded drug	Application disease	Refs.
ligand‐receptor interactions	1	E‐selectin‐modified thermosensitive nanomicelles	E‐selectin‐modified thermosensitive nanomicelles	Leukocytes	E‐selectin	Glycoprotein on leukocyte surface	Doxorubicin (DOX), A2A adenosine receptor antagonist (SCH 58 261)	Induce immunogenic cell death of tumors, relieve immune suppression, inhibit tumor growth, anti‐metastasis, and anti‐recurrence	Tumor	[44]
	2	ES‐GBL	E‐selectin‐modified liposomal nanoparticles	Leukocytes	E‐selectin	Sialylated carbohydrates on leukocyte surface	Gemcitabine (GemE) and PD‐1 inhibitor BMS‐202	Induce pyroptosis of HCC cells, promote immune response, inhibit immune escape, significantly enhance the efficacy of liver cancer immunotherapy	Tumor (hepatocellular carcinoma)	[40]
	3	APT	Ac‐PGP peptide‐modified tetrahedral framework nucleic acid nanoparticles	Neutrophils	Ac‐PGP peptide sequence	CXCR2 receptor	All‐trans retinoic acid (ATRA)	Induce differentiation of promyelocytes into mature granulocytes, inhibit the proportion and absolute number of neutrophils	Sepsis	[45]
	4	HPNFcN	Sialic acid (SA)‐modified semiconducting polymer nanoparticles	Neutrophils	Sialic acid (SA)	None	Ferroptosis inducer (ferrocene prodrug) and immunotherapy drug NLG919	Induce ferroptosis, trigger immunogenic cell death, enhance antitumor immune response	Glioblastoma	[46]
	5	T‐TMP	CFLFLF peptide‐modified PLGA nanoparticles	Neutrophils	CFLFLF peptide	FPR receptor	Ligustrazine	Reduce reperfusion injury	Ischemic stroke	[47]
	6	H‐ANEs	Selenium (Se)‐containing nano‐integrated cascade enzymes (h‐ANEs) co‐hybridized with catalase (CAT), superoxide dismutase 1 (SOD1), and bovine serum albumin (BSA)	Neutrophils	‐	Fcγ receptor	Catalase (CAT), superoxide dismutase (SOD1), selenium (Se)	Antioxidation and anti‐ferroptosis, reduce reperfusion injury	Ischemic stroke	[48]
	7	D@MLL	Nanomaterial modified with lipid MMP‐2 responsive peptide, lipoteichoic acid (LTA)	Monocytes	Lipoteichoic acid (LTA)	CD14 receptor	Doxorubicin (DOX·HCl)	Induce immunogenic cell death, promote antitumor immune response	Glioblastoma	[49]
	8	MCP1‐Gd Micelles	Micelles modified with MCP1	Monocytes	Peptide with CCR2 binding motif (MCP1)	CCR2 receptor	Gadolinium (Gd)	Enhance MRI signal, detect metastatic LN	Metastatic lymph nodes	[41]
	9	SHIDS	Nanoparticles modified with modified chitosan	Red blood cells	Aminoglucose on chitosan	GLUT	Insulin, glucose oxidase (GOx), catalase (CAT)	Closed‐loop glucose regulation, achieve long‐term automatic blood glucose control	Diabetes	[42]
	10	CR‐TNG	Nanoparticles modified with anti‐cd47 antibody (aCD47)	Leukemia cells	Anti‐TIM‐3 antibody	TIM‐3 protein	Resiquimod (R848)	Block CD47, activate immune response	Acute myeloid leukemia	[50]
	11	C&H@MOF/PL‐A	Metal‐organic framework (MOF) nanoparticles modified with PD‐1 antibody	PD‐1⁺ T Cells	PD‐1 antibody (aPD‐1)	PD‐1	Hemoglobin (Hb) and catalase (CAT)	Relieve tumor hypoxia, activate immune response, inhibit tumor growth, anti‐metastasis, and anti‐recurrence	Colorectal cancer	[43]

#### 2.1.2 “Backpack” Strategy by Covalent Binding

Covalent conjugation refers to the stable bonding of NPs to specific molecules on the cell surface through chemical reactions. This approach enhances the stability of NPs on the cell surface, reducing detachment caused by blood shear forces. However, covalent conjugation involves complex synthetic procedures and may alter the structure and function of the cell membrane, potentially affecting cellular physiological properties. This method is commonly applied to endogenous biological carriers such as red blood cells (RBCs) and albumin (**Table** **3**).

**Table 3:** “Backpack” strategy by covalent binding.

Loading strategy	No.	Nanomedicine name	Nanomedicine structure	Hitchhiking object	Key substance	Loaded drug name	Function of loaded drug	Application disease	Refs.
Covalent binding	1	IR1080	H‐aggregate and albumin covalently bonded	Albumin	‐	Near‐infrared window II (NIR‐II) fluorescent probe IR1080	Improve detection rate and edge delineation ability, enhance the contrast between tumor and normal tissue	Tumor micrometastasis	[51]
	2	Nutri‐hijacker	Albumin covalently modified by biguanide drugs and flavonoids	Albumin	‐	Biguanide drugs, flavonoids	Biguanide drugs inhibit glycolysis, flavonoids inhibit glutaminolysis	Pancreatic ductal adenocarcinoma	[53]
	3	EB‐ss‐DM1	Evans blue (EB) and maytansine (DM1) connected through a responsive disulfide bond; EB covalently bonded with albumin	Albumin	‐	Evans blue (EB) and maytansine (DM1)	Cytotoxic effect, induce tumor cell death	Tumor	[52]
	4	SS31‐Rapa	Conjugate nanoparticles (SS31‐Rapa) formed by a cleavable linker between SS31 (mitochondria‐targeted antioxidant tetrapeptide) and rapamycin (autophagy inducer)	Red blood cells	FK506‐binding protein (FKBP)	SS31 (mitochondria‐targeted antioxidant tetrapeptide) and rapamycin (autophagy inducer)	Induce autophagy	Acute kidney injury (AKI)	[54]
	5	MSCFM	Janus mesenchymal stem cells loaded with melanin nanoparticles	Mesenchymal stem cells	Tannic acid (TA)	Melanin nanoparticles (FM) formed by polymerization of dopamine and iron ions	Antioxidation, free radical scavenging, immune response regulation	Rheumatoid arthritis	[55]
	6	AZCE‐MN	Functionalized gold@cerium‐zinc composite core‐shell nanoparticles (AZC‐A) were combined with Escherichia coli (E. coli). Subsequently, AZCE was combined with microneedle carriers to prepare AZCE‐MN	Escherichia coli (E. coli)	Amino (‐NH₂) group	Gold@cerium‐zinc composite	(1) Zn^2^⁺ induces mitochondrial damage and enhances the production of reactive oxygen species (ROS). (2) The redox cycle between Ce^3^⁺/Ce⁴⁺ can effectively consume glutathione (GSH). (3) Under near‐infrared (NIR) laser irradiation, Au nanorods can produce photothermal effects	Triple‐negative breast cancer	[56]

In the context of albumin, leveraging tumor microenvironment‐specific proteins (e.g., gp60 receptors and SPARC) that interact with albumin, Xu et al. designed an NIR‐II window fluorescent probe, IR1080, which switches fluorescence from an “off” to an “on” state upon covalent conjugation with albumin. This covalent interaction enables precise detection of micro‐metastases (<2 mm), facilitating surgical navigation.^[^ ^51^ ^]^ Building on the metabolic dependence of pancreatic ductal adenocarcinoma (PDAC) with KRAS gene mutations on albumin uptake, Fu et al. developed Evans blue (EB)‐conjugated NPs (EB‐ss‐DM1) by covalently linking EB (which binds albumin) with the highly cytotoxic drug DM1. These NPs exploit albumin's transport function to deliver DM1 to tumors, inhibiting tumor growth.^[^ ^52^ ^]^ Similarly, Huang et al. engineered a “Nutri‐hijacker” nanomaterial that covalently binds albumin. Upon entering KRAS‐mutant cancer cells via macropinocytosis, it reprograms tumor metabolism using biguanides and flavonoids.^[^ ^53^ ^]^ For red blood cells, Yu et al. synthesized NPs conjugates (SS31‐Rapa) by combining SS31 with rapamycin (an autophagy inducer). These NPs specifically bind to the FK506‐binding protein (FKBP) within RBCs, enabling targeted delivery to the intracellular space of RBCs. This strategy extends the half‐life of SS31 by 6.9‐fold and reverses cisplatin‐ or ischemia‐reperfusion‐induced acute kidney injury through autophagy induction.^[^ ^54^ ^]^

#### 2.1.3 “Backpack” Strategy by Non‐Covalent Binding

Non‐covalent binding primarily includes hydrophobic interactions, electrostatic interactions, hydrogen bonding and van der Waals forces, and host–guest interactions. These interactions mainly occur in non‐endocytic or poorly endocytic circulatory carriers and are widely employed in endogenous carriers such as RBCs, albumin, immunoglobulins, and platelets (**Table** **4**). However, non‐covalent strategies currently face several challenges: 1) Maintaining stability under circulatory shear forces: The binding affinity between NPs and circulatory carriers must be sufficiently strong to withstand shear forces in the bloodstream. 2) Achieving efficient release in target tissues: NPs need to be successfully released upon reaching the target tissue; otherwise, their functionality may be compromised. 3) Optimizing factors influencing specific adsorption: This includes tuning NPs surface charge, particle size, and shape to ensure robust non‐covalent binding.

**Table 4:** “Backpack” strategy by non‐covalent binding.

Loading strategy	No.	Nanomedicine name	Nanomedicine structure	Hitchhiking cells	Key substance	Loaded drug name	Function of loaded drug	Application disease	Refs.
Non‐covalent binding	1	OPDEA‐PS	OPDEA‐based nanomicelles	Red blood cells	‐	Clarithromycin	Antibacterial effect	Pneumonia	[58]
	2	Dox‐IONPs	Nanoparticles based on multi‐particle iron oxide (MIO)	Red blood cells	‐	Doxorubicin (Dox)	Under the action of an alternating magnetic field, multi‐particle iron oxide (MIO) generates heat, thereby releasing tumor‐associated antigens	Tumor pulmonary metastasis	[29]
	3	MPSS‐CSNPs	Chitosan nanoparticles	Red blood cells	‐	Methylprednisolone succinate sodium (MPSS)	Anti‐inflammatory effect of the hormone	COVID‐19 pneumonia	[33]
	4	G‐OVA‐PLGA	β‐glucan‐ovalbumin (OVA) complex nanoparticles encapsulated by PLGA	Red blood cells	‐	β‐glucan and ovalbumin (OVA)	1) β‐glucan promotes macrophage activation towards the M1 type; 2) Ovalbumin (OVA) as a model antigen can be taken up and processed by antigen‐presenting cells to activate T cells	COVID‐19 pneumonia	[34]
	5	SIM‐PEI‐PPNPs	pH‐responsive cationic simvastatin nanoparticles	Red blood cells	‐	Simvastatin	Alleviate acute respiratory distress syndrome	Acute respiratory distress syndrome (ARDS)	[35]
	6	IVM‐PNPs; IVM‐CSPNPs	Ivermectin nanoparticles encapsulated by PLGA or chitosan	Red blood cells	‐	Ivermectin	Antiviral and anti‐inflammatory effects	COVID‐19 pneumonia	[36]
	7	β‐cyclodextrin‐modified ferene liposomes	Liposomes loaded with curcumin modified by ferene (Fc)	Red blood cells	β‐cyclodextrin	Curcumin	Anti‐inflammatory effect	Acute pneumonia	[30]
	8	PC, PY	Paclitaxel prodrug conjugated with fatty acid chains	Albumin	‐	Paclitaxel	Antitumor	Tumor	[31]
	9	rTCS‐PTN‐ABD	Fusion protein integrating albumin‐binding domain (ABD), legumain (a cysteine protease), and trichosanthin (TCS)	Albumin	Albumin‐binding domain (ABD)	Trichosanthin (TCS)	Inhibit tumor cell protein synthesis	Tumor	[37]
	10	FA‐sLip/OVA/MPLA	Liposomes modified by folic acid (FA)	Natural IgM	Folic acid	1) Ovalbumin (OVA) as a tumor model antigen; 2) TLR4 agonist (MPLA) activates the immune response	Activate B cells to produce antibodies and enhance immune response	Tumor	[32]
	11	TDHP	Dual‐mode biosensor based on PNA/peptide copolymer and DNA tetrahedron	Macrophages	DSPE‐PEG200	Multisegment PNA/peptide copolymer (4PD)	Tumor imaging and urine analysis	Tumor	[38]
	12	NLMP2	Peptide nanofibers	Platelets	No ligand modification	‐	Pulmonary‐targeted drug delivery	Pneumonia	[39]

For RBC‐based applications, Liang et al. designed doxorubicin (Dox)‐loaded multi‐particle iron oxide NPs (Dox‐IONPs). First, RBCs were extracted from the body and co‐incubated with the NPs. Through osmotic shock‐mediated fusion, the multi‐particle iron oxide (MIO) was assembled onto the RBC surface. Reversible interactions allowed the MIO to detach from RBCs under shear stress in pulmonary microvessels following intravenous injection, thereby transferring MIO to lung capillary endothelial cells for subsequent enrichment in tumor tissues.^[^ ^29^ ^]^ Li et al. utilized β‐cyclodextrin (β‐CD)‐modified RBCs as hosts and ferrocene (Fc)‐functionalized curcumin‐loaded liposomes as guests. By leveraging host–guest interactions, the NPs were attached to RBCs in vitro for RBC‐mediated transport. Upon reaching inflammatory sites, curcumin was selectively released under ROS stimulation, effectively alleviating acute pneumonia symptoms.^[^ ^30^ ^]^ For albumin‐based strategies, Lu et al. modified paclitaxel (PTX) with single‐chain and double‐chain fatty acids, forming self‐assembled PC and PY NPs, respectively. These NPs non‐covalently bound to human serum albumin (HSA), forming long‐circulating nanocomplexes (PC/HSA NCs and PY/HSA NCs). Albumin transported PTX‐loaded NPs into tumor cells for therapeutic efficacy.^[^ ^31^ ^]^ Regarding immunoglobulin applications, prior studies revealed that folate (FA) ‐modified liposomal NPs (FA‐sLip/OVA/MPLA) was designed to “hitchhike” on blood‐circulating IgM for spleen delivery. This system co‐delivered the tumor model antigen ovalbumin (OVA) to splenic marginal zone B cells (MZB cells) and triggered immune responses as an immunoadjuvant.^[^ ^32^ ^]^

### 2.2 Encapsulation‐Mediated “Trojan Horse” Strategy

The “Trojan horse” strategy mediated by encapsulation, which involves encapsulating drugs or drug carriers within cells through methods such as hypotonic dialysis, endocytosis, or electroporation, offers advantages including protection of intracellular drugs or carriers from serum enzyme metabolism and off‐target toxicity, enhanced stability and targeting efficiency by preserving cell membrane integrity to prevent NPs detachment, and operational simplicity and controllability. However, challenges persist, such as intracellular degradation of NPs due to hydrolytic enzymes and reactive oxygen species (ROS), risks of off‐target drug release via macrophage exocytosis, and potential functional interference with immune cells (e.g., altered macrophage phenotype/activation) that may compromise immunotherapy.^[^ ^73^ ^]^ This strategy is primarily applied to phagocytic immune cells like neutrophils, monocytes/macrophages, and mesenchymal stem cells (MSCs), with this section focusing on the encapsulation mechanisms of neutrophils and macrophages (**Table** **5**).

**Table 5:** “Trojan horse” strategy by neutrophils and monocytes/macrophages.

Hitchhiking pathway	No.	Nanomedicine name	Nanoscale structure	Hitchhiking cells	Ligands on nanomedicine	Receptors on hitchhiking cells	Loaded drug name	Function of loaded drug	Application disease	Refs.
Ligand‐receptor‐mediated encapsulation	1	NPs@NEs	PLGA nanoparticles modified with cRGD peptide	Neutrophils	cRGD peptide	L‐selectin	Cabazitaxel, dexamethasone, teriparatide	Antitumor or anti‐osteoporosis	Bone metastatic cancer or osteoporosis	^[^ ^57^ ^]^
	2	cRGD‐modified dexamethasone liposomes	Liposomal nanoparticles modified with cRGD peptide	Neutrophils	cRGD peptide	L‐selectin	Dexamethasone	Anti‐inflammatory effect, immune response regulation	Sepsis	^[^ ^58^ ^]^
	3	cRGD‐SVT‐Lipo	Liposomal nanoparticles modified with cRGD peptide	Neutrophils	cRGD peptide	Integrin ανβ3	None	Inhibits neutrophil elastase activity, reduces plaque inflammation and destruction	Atherosclerosis	[59]
	4	c(RGDfk)‐functionalized liposomes	Liposomal nanoparticles modified with cRGD peptide	Neutrophils	cRGD peptide	Integrin	Carfilzomib and BMS‐202	Immune regulation, enhanced antitumor immune response	Multiple myeloma	[60]
	5	SPPS	Nanoparticles modified with sialic acid (SA)	Neutrophils	Sialic acid (SA)	L‐selectin	siBcl‐2 gene drug, polydopamine (PDA)	Blocks NETosis, increases bacterial survival in tumors	Tumor	^[^ ^61^ ^]^
	6	D@HPB@SPM NPs	Hollow prussian blue nanoparticles core with DNase I loaded and sialic acid (SA)‐modified platelet membrane shell	Neutrophils	Sialic acid (SA)	αMβ2 integrin	Deoxyribonuclease I (DNase I)	Clears ROS, degrades NETs	Ischemic stroke	^[^ ^62^ ^]^
	7	n‐DOCPs	Drug‐loaded liposomal nanoparticles	Neutrophils	Complement fragment iC3b (attached during circulation)	CR3 receptor	Dexamethasone (Dex), antibiotics (e.g., Ampicillin)	Bacterial killing	Acute lung injury	[63]
	8	CPPC	Polydopamine (PDA) nanoparticles with anti‐CD11b antibody	Neutrophils	Anti‐CD11b antibody	CD11b antigen	Antibiotics, cordycepin (Cor)	Anti‐inflammatory effect	Acute lung injury	^[^ ^64^ ^]^
	9	Anti‐Ly6G antibody‐modified liposomes	Liposomes modified with anti‐Ly6G antibody	Neutrophils	Anti‐Ly6G antibody	Ly6G	Anticancer drug doxorubicin (DOX) and non‐nucleoside STING agonist SR‐717	Inhibits tumor recurrence and metastasis	Tumor	^[^ ^65^ ^]^
	10	APTB	Tetrahedral framework nucleic acid nanoparticles modified with Ac‐PGP peptide	Neutrophils	Ac‐PGP peptide sequence	CXCR2 receptor	Baicalin	Inhibits neutrophil inflammatory response, promotes macrophage polarization	Inflammatory diseases	[45]
	11	BSA‐GOx‐NPs	Bovine serum albumin nanoparticles loaded with glucose oxidase (GOx)	Neutrophils	Fcγ receptor on neutrophil surface	Fcγ receptor on neutrophils	GOx	Catalyzes glucose to produce gluconic acid and hydrogen peroxide, induces cell apoptosis by depleting glucose	Endometriosis	[66]
	12	Liposomal nanoparticles modified with cRGD peptide	Liposomal nanoparticles modified with cRGD peptide	Monocytes/Macrophages	cRGD peptide	Integrin ανβ3	‐	Tumor targeting	Tumor	^[^ ^60^ ^]^
	13	DA‐βGlus/ODs/PTX	Nanoparticles modified with deaminated glucosaminoglycan (DA‐βGlus)	Monocytes/Macrophages	Deaminated glucosaminoglycan (DA‐βGlus)	Dectin‐1 receptor	Paclitaxel (PTX)	Reduces tumor stromal fibrosis, precisely delivers paclitaxel (PTX), inhibits tumor growth	Pancreatic cancer	^[^ ^67^ ^]^
	14	𝜷Glus‐ZnD	Nanoparticles modified with β‐cyclodextrin	Monocytes/Macrophages	β‐cyclodextrin (βGlus)	Macrophage βGlus receptor	Chemotherapy drug Doxorubicin (DOX) and zinc ions (Zn^2^⁺)	DOX induces tumor cell apoptosis, Zn^2^⁺ enhances therapeutic effect	Pancreatic cancer	[68]
	15	bPEI‐SS‐PEG‐T/NLS/DNA	Peptide segment with nuclear localization signal (NLS) and polymer nanoparticles crosslinked with tuftsin‐modified disulfide bonds	Monocytes/Macrophages	Tuftsin	Macrophage tuftsin receptor	IL‐10 plasmid DNA	Regulates macrophage metabolic reprogramming, inhibits mTOR activity, induces apoptosis	Rheumatoid arthritis	[69]
	16	SH‐β CD; SH‐Fc	(1) Copper sulfide nanoparticles modified with β‐cyclodextrin (SH‐β CD); (2) Copper sulfide nanoparticles modified with bipyridine (SH‐Fc)	Monocytes/Macrophages	‐	‐	Copper sulfide (CuS)	Penetrates tumor tissue, produces photothermal effect, induces tumor cell apoptosis	Tumor	[70]
	17	Citop‐NMs	Nanomicelles based on amphiphilic polymer DSPP	Monocytes/Macrophages	‐	‐	Citral and sulfo‐captopril	Reduces NLRP3‐dependent IL‐1β secretion, lowers blood pressure, and has anti‐atherosclerotic effects	Atherosclerosis	[71]
	18	QD‐Ce6	Nanoparticles composed of photoluminescent quantum dots (QDs) and photosensitizer chlorophyll e6 (Ce6)	Mesenchymal stem cells	‐	‐	QDs, Ce6	Ce6 generates reactive oxygen species, disrupts cancer cell structure, achieves photodynamic therapy	Tumor	[72]

#### 2.2.1 “Trojan Horse” Strategy by Neutrophils

To achieve neutrophil‐mediated endocytosis, nanomaterials are often functionalized with ligands such as cyclic arginine‐glycine‐aspartic acid (cRGD) peptides, sialic acid (SA), Ac‐PGP peptides, and glucose oxidase (GOx). For instance, leveraging the interaction between αvβ3 integrins on neutrophil surfaces and cRGD peptides, cRGD‐modified PLGA NPs (NPs@NEs) are internalized by neutrophils, traverse the bone marrow‐blood barrier, and release cabazitaxel at lesion sites to inhibit bone metastasis growth.^[^ ^57^ ^]^ Similarly, SA‐decorated NPs (SPPS) bind to neutrophil L‐selectin and αMβ2 integrins, enabling their uptake. Upon migration to tumor regions, SPPS releases polydopamine (PDA) to block neutrophil extracellular trap (NET) formation and delivers siRNA (siBcl‐2) to enhance tumor cell apoptosis.^[^ ^61^ ^]^ For C‐X‐C motif chemokine receptor 2 (CXCR2) receptor‐targeting, Ac‐PGP peptide‐modified tetrahedral framework nucleic acid NPs (APTB) specifically recognize neutrophil CXCR2 receptors, facilitating their uptake and transport to inflammatory sites, where baicalin is released to exert anti‐inflammatory effects.^[^ ^45^ ^]^ Additionally, GOx‐conjugated bovine serum albumin NPs (BSA‐GOx‐NPs) exploit Fcγ receptor binding on neutrophils for encapsulation. These NPs are transported to endometriosis lesions, where GOx depletes glucose to induce apoptosis in ectopic endometrial cells.^[^ ^66^ ^]^

Based on the presence of antigens such as CD11b and Ly6G on the surface of neutrophils, nanomaterials can also be functionalized with antibodies to bind to the antigens on the surface of white blood cells, thereby achieving encapsulation. To bind to the CD11b antigen on the surface of neutrophils, anti‐CD11b antibodies are conjugated to the surface of PDA NPs (CPPC). After phagocytosis by neutrophils, the NPs are transported to the area of acute lung injury, where cordycepin is released to exert its anti‐inflammatory effects.^[^ ^64^ ^]^ To bind to the Ly6G antigen on the surface of neutrophils, the specific binding of biotin and streptavidin is utilized to attach anti‐Ly6G antibodies to the surface of liposomes, mediating phagocytosis by neutrophils. Due to the chemotaxis of neutrophils to tumor areas, nanomaterials can release the anticancer drugs doxorubicin (DOX) and the non‐nucleoside stimulator of interferon genes (STING) agonist SR–717 in tumor areas through NETs, thereby inhibiting tumor recurrence and metastasis.^[^ ^65^ ^]^

#### 2.2.2 “Trojan Horse” Strategy by Monocytes/Macrophages

Monocytes/macrophages, as the most common phagocytes, often serve as “Trojan horses” in targeted nanomedicine delivery, enabling spatiotemporally controlled drug release and activation at target sites. For instance, leveraging the interaction between Dectin‐1 receptors on monocyte/macrophage surfaces and β‐glucan (βGlus), orally administered DA‐βGlus/ODs/PTX NPs are phagocytosed by macrophages via Dectin‐1 binding, transported to pancreatic cancer regions, and release PTX to reduce fibrosis.^[^ ^67^ ^]^ Similarly, βGlus‐modified NPs (βGlus‐ZnD) are internalized by endogenous macrophages post‐oral administration through βGlus receptor binding. Driven by homing effects, these NPs accumulate in tumors and release DOX to induce tumor cell apoptosis.^[^ ^68^ ^]^ Interestingly, monocytes/macrophages not only act as “hitchhiking” carriers but also allow NPs to modulate their phenotype and functional reprogramming. For example, tuftsin receptor‐targeted NPs (bPEI‐SS‐PEG‐T/NLS/DNA) bind to tuftsin receptors on macrophages, mediating encapsulation and transport to inflammatory sites, where they induce apoptosis.^[^ ^69^ ^]^

## 3 Different Biological Carriers for “Hitchhiking” Strategy

The integration of NPs with biological carriers holds immense promise for targeted drug delivery and imaging in nanomedicine applications. However, ensuring the safe and effective use of this approach critically depends on selecting carriers with suitable properties. In this section, we briefly introduce cellular biological carriers (e.g., white blood cells such as neutrophils and macrophages, and red blood cells) and acellular biological carriers (e.g., albumin and bacteria). The advantages and disadvantages of different biological carriers are listed in Table S1 (Supporting Information).

### 3.1 Cellular Biological Carriers

#### 3.1.1 Neutrophils‐Based BCM‐NHS

Neutrophils, constituting 50–70% of white blood cells, are the first cells to arrive at inflammatory sites and the most abundant cells recruited during acute inflammation. Their potent antimicrobial activity and phagocytic capacity make them highly efficient drug carriers.^[^ ^77^, ^78^, ^79^ ^]^ While traditionally considered short‐lived (6–8 h), recent studies suggest a circulatory lifespan of ≈5.4 days in humans.^[^ ^80^ ^]^ Neutrophils participate in immune responses through phagocytosis, NETs release, and ROS production.^[^ ^73^, ^79^, ^81^ ^]^ Ex vivo engineering strategies are limited by neutrophils’ short lifespan, premature intracellular cargo degradation, high costs, insufficient cell yield, and contamination risks, making in vivo hitchhiking approaches more clinically viable.^[^ ^82^ ^]^ Here are several advantages for neutrophil‐based BCM‐NHS: 1) Immune evasion: Neutrophils membranes inherit source cells' self‐recognition mechanisms and natural immune receptors, enabling nanoparticles to evade immune clearance; 2) Inflammation and tumor targeting: The surface expression of chemokine receptors and other innate immune recognition receptors allows for active migration to inflammatory or tumor sites, ensuring precise targeting. 3) Transendothelial migration capability: The high membrane fluidity and natural endothelial migration ability of neutrophils enable them to carry nanoparticles across vascular barriers, overcoming biological obstacles that impede conventional delivery systems. However, challenges for neutrophil‐based BCM‐NHS also remain: 1) Short neutrophil lifespan: premature drug release risks off‐target accumulation and toxicity; 2) Drug loading and stability: limited cargo capacity and intracellular drug instability require refinement^[^ ^79^ ^]^; 3) Functional interference: excessive neutrophil activation or accumulation may cause tissue damage. Thus, precise control over drug release kinetics and minimizing unintended neutrophil modulation are critical for successful neutrophil‐based BCM‐NHS.^[^ ^73^ ^]^

Neutrophil‐based BCM‐NHS primarily relies on ligand‐receptor‐mediated “backpack” strategies or “Trojan horse” mechanisms (Table S2, Supporting Information). Leveraging surface targets such as L‐selectin, integrins, CXCR2 receptors, Fcγ receptors, complement receptor 3 (CR3), CD11b, and Ly6G antigens, NPs functionalized with corresponding ligands achieve neutrophil‐mediated drug transport. This strategy has been applied in ischemic stroke, atherosclerosis, inflammation, cancer, endometriosis, and renal fibrosis.

For ischemic stroke treatment, Trac et al. synthesized nanocascade enzymes (h‐ANEs) via a one‐pot method. These nanozymes bind to Fcγ receptors on neutrophil surfaces, leveraging neutrophil inflammation tropism to cross the blood‐brain barrier. They release CAT and superoxide dismutase 1 (SOD1) to scavenge ROS through cascade reactions, while c (Se) enhances glutathione peroxidase 4 (GPX4) expression, inhibiting neuronal ferroptosis to treat ischemia‐reperfusion (I/R)‐induced brain injury.^[^ ^48^ ^]^ For atherosclerosis, Shi et al. utilized cRGD‐modified liposomal NPs (cRGD‐SVT‐Lipo) that “hitchhike” neutrophils to atherosclerotic sites, releasing the drug SVT to suppress NETs formation and reduce plaque inflammation.^[^ ^59^ ^]^ In inflammation therapy, Zhou et al. developed Ac‐PGP peptide‐modified tetrahedral framework nucleic acid NPs (APT). By binding neutrophil CXCR2 receptors via surface Ac‐PGP, APT attaches to neutrophils and targets inflammatory regions via chemotaxis. The NPs release all‐trans retinoic acid (ATRA), reducing neutrophil counts and oxidative bursts to mitigate sepsis‐induced tissue damage.^[^ ^83^ ^]^ For tumor therapy, sialic acid (SA)‐conjugated semiconducting polymer NPs (HPNFcN) hitchhike neutrophils to glioblastoma sites, releasing ferroptosis inducers and the immunotherapeutic drug NLG919. Under X‐ray irradiation, HPNFcN induces tumor cell ferroptosis and triggers immunogenic cell death.^[^ ^46^ ^]^

#### 3.1.2 Monocytes/Macrophages‐based BCM‐NHS

Monocytes are precursors of macrophages that can migrate from the blood through endothelial cell barriers to the depths of each organ and differentiate into macrophages there. Macrophages can phagocytose and clear foreign substances in the blood and also migrate to inflammatory sites or tumor tissues. Here are several advantages for monocytes/macrophages‐based BCM‐NHS: 1) Immune evasion: Due to their natural expression of “self”‐markers, monocytes/macrophages evade clearance by the RES, thereby prolonging their circulation time and enhancing drug delivery efficiency; 2) Inflammation and tumor targeting: Equipped with diverse surface receptors (e.g., chemokine receptors, scavenger receptors), these cells can precisely home in on inflammatory or tumor microenvironments. Their innate phagocytic capacity and migratory properties enable targeted drug delivery even to poorly accessible regions, such as hypoxic tumor cores.^[^ ^84^, ^85^, ^86^, ^87^, ^88^, ^89^, ^90^, ^91^ ^]^ 3) Transendothelial migration capabilities: A critical advantage is their ability to cross tightly regulated biological barriers, including the blood‐brain barrier (BBB) and bone marrow‐blood barrier, facilitating therapeutic delivery to shielded tissues. 4) Immune microenvironment modulation: Membrane proteins from differently polarized monocytes/macrophages and neutrophils can interact with immune cell receptors to regulate local immune status, providing dual therapeutic strategies. The primary limitations for monocytes/macrophages based‐ BCM‐NHS include limited extraction yield and potential risks of immune overresponse.

The complexity of isolation and cultivation procedures increases therapeutic costs through in vitro synthesis, making the “in situ hitchhiking strategy” of macrophages more widely applicable. The integration methods of nanomaterials with monocytes/macrophages include surface attachment and encapsulation. Notably, in surface attachment designs, larger‐diameter NPs (often disk‐shaped) are required to avoid encapsulation by macrophages. The binding of macrophages to NPs primarily relies on ligand‐receptor interactions and encapsulation, with occasional non‐covalent binding (Table S3, Supporting Information). Based on the common presence of Dectin‐1 receptors, CD14 receptors, CCR2 receptors, Fcγ receptors, βGlus receptors, and tuftsin receptors on monocyte/macrophage surfaces, previous studies have frequently used corresponding ligands to modify NPs for ligand‐receptor‐mediated “hitchhiking” strategies.

Monocytes/macrophages are mainly applied in tumors, rheumatoid arthritis, and arterial fields. For tumor treatment, Kuang et al. utilized LTA‐modified nanomaterials (D@MLL) that bind to CD receptors on monocyte surfaces, enabling attachment to monocytes. Leveraging monocyte migration capabilities to cross the BBB, they released doxorubicin hydrochloride (DOX·HCl) to induce immunogenic cell death and promote anti‐tumor immune responses.^[^ ^49^ ^]^ Li et al. designed two copper sulfide (CuS) NPs: β‐cyclodextrin‐modified SH‐β‐CD and bipyridine‐modified SH‐Fc NPs. After being phagocytosed by macrophages, these NPs formed supramolecular aggregates intracellularly during macrophage‐mediated “hitchhiking” delivery, inhibiting CuS NPs efflux. Upon reaching tumor areas, copper sulfide (CuS) was released to achieve deep tissue penetration and enhanced photothermal therapy.^[^ ^70^ ^]^ For atherosclerosis treatment, Fu et al. developed an amphiphilic polymer DSPP‐based nanomicelle (Citop‐NMs) that is phagocytosed by monocytes and transported to atherosclerotic plaques. Monocytes differentiate into foam cells in plaques, where the massive release of ROS stimulates NPs release of citral and thio‐captopril to suppress inflammatory responses.^[^ ^71^ ^]^ Regarding rheumatoid arthritis therapy, Zhang et al. designed NPs (bPEI‐SS‐PEG‐T/NLS/DNA) that bind to tuftsin receptors on macrophages, mediating macrophage encapsulation of NPs for transport to inflammatory regions. Within macrophage nuclei, IL‐10 plasmid DNA (IL‐10 pDNA) is released and translocated to the nucleus via nuclear localization signal (NLS)‐containing peptides in the NPs, undergoing transcription and translation to regulate macrophage metabolic reprogramming.^[^ ^69^ ^]^

#### 3.1.3 Red blood cells‐based BCM‐NHS

Red blood cells (RBCs) are the most abundant cell type in mammals, with a diameter of approximately 7–8 µm, and can be easily isolated via simple centrifugation. Due to their unique biological properties, RBCs serve as excellent carriers for BCM‐NHS, offering several key advantages (Table S4, Supporting Information): 1) Immune evasion: RBCs express CD47 protein, a “self‐marker” that acts as a cellular “passport,” enabling immune evasion by inhibiting phagocytosis,^[^ ^92^, ^93^, ^94^, ^95^, ^96^, ^97^ ^]^ while membrane proteins such as CR1, C8bp, and CD59 further suppress complement activation, reducing nanoparticle immunogenicity.^[^ ^98^ ^]^ 2) High abundance and easy extraction: As the most prevalent blood cells, RBCs possess a mature, enucleated structure that allows straightforward membrane isolation and purification, making them ideal for nanocarrier development.^[^ ^99^ ^]^ 3) Extended circulation time: RBCs exhibit remarkable longevity with a lifespan of 100–120 days.^[^ ^100^ ^]^ 4) High deformability and flexibility: Mechanically, their high flexibility enables reversible deformation when passing through capillaries smaller than their diameter. The most extensively studied target organ is the lungs.^[^ ^101^ ^]^ 5) Structural simplicity: RBCs possess a unique biconcave disk shape, enucleated structure, and high surface area‐to‐volume ratio, facilitating efficient drug loading. The primary limitations of RBC membranes include their lack of intrinsic targeting capability and the need for blood type matching. To address this, researchers have employed targeted peptide modifications or hybrid membrane strategies with other cell types to confer tumor‐ or inflammation‐specific targeting.^[^ ^102^ ^]^ Drug‐loading methods for RBCs similarly include surface attachment and internalized encapsulation,^[^ ^101^, ^103^ ^]^ with non‐covalent binding being the most common approach for surface attachment.^[^ ^25^, ^101^, ^104^ ^]^ The primary limitations of RBC‐based BCM‐NHS include their lack of intrinsic targeting capability and the need for blood type matching.

RBC‐based delivery technologies are primarily applied in disease models such as pneumonia, diabetes, and tumors. For pneumonia treatment, Yu et al. developed OPDEA‐PS micelles, leveraging their RBC membrane affinity and Gram‐positive bacterial cell wall‐binding capability to prolong circulation time, enabling precise release of clarithromycin at infection sites.^[^ ^105^ ^]^ Ding et al. designed methylprednisolone sodium succinate (MPSS)‐loaded chitosan NPs (MPSS‐CSNPs). After extracting RBCs from the body, the NPs were co‐incubated with RBCs to non‐covalently load hormones onto RBC surfaces (RBC‐MPSS‐CSNPs), reducing liver uptake of NPs, promoting pulmonary hormone accumulation, and minimizing steroid dependency and severe side effects caused by high‐dose steroid use in COVID‐19 pneumonia therapy.^[^ ^33^ ^]^ For diabetes management, Li's team constructed glucosamine‐modified chitosan NPs (SHIDS) to achieve RBC‐targeted anchoring via amino‐GLUT receptor interactions. This system utilizes glucose oxidase (GOx) to catalyze gluconic acid/H₂O₂ production, establishing an acidic microenvironment that triggers pH‐responsive chitosan conformational changes to release insulin. Upon blood glucose normalization, microenvironmental pH restoration halts drug release, forming a biomimetic closed‐loop regulatory mechanism that precisely mimics pancreatic β‐cell secretion dynamics.^[^ ^42^ ^]^ For tumor immunotherapy, Liang's group developed doxorubicin‐iron oxide nanocomposites (Dox‐IONPs) delivered intravenously via RBC surface fusion. Magnetothermal therapy triggered tumor antigen release, which was cross‐presented by dendritic cells to lymph nodes, significantly prolonging survival in lung cancer metastasis models.^[^ ^29^ ^]^

Current challenges for RBC carriers include: 1) NPs shape, surface charge, size, and hydrophilicity/hydrophobicity may induce RBC morphological changes, potentially causing hemolysis or aggregation. For example, extensive nanodiamond attachment significantly alters RBC morphology into an echinocyte form.^[^ ^104^ ^]^ 2) Due to lacking phagocytic capacity, RBC intracellular drug loading often involves membrane‐disruptive methods, with hemoglobin release from damaged RBCs posing risks of kidney failure. 3) Compared to immune cells, RBCs exhibit limited ability to cross biological barriers. Thus, RBC‐based drug delivery requires comprehensive consideration of loading methods, drug payload, disease applicability, and other factors.

#### 3.1.4 Platelets‐based BCM‐NHS

Platelets are anucleate discoid cell fragments (2‐3 µm diameter) derived from megakaryocyte cytoplasm, playing pivotal roles in hemostasis, inflammation, and immune regulation.^[^ ^106^ ^]^ Their unique biological properties make them promising biomimetic nanocarrier materials: 1) Immune evasion: Platelets exhibit excellent immune escape capability with relatively long circulation time (8–9 days)^[^ ^107^ ^]^;CD47 molecules and complement inhibitory proteins (CD55, CD59) synergistically reduce macrophage phagocytosis and complement attack, prolonging nanoparticle circulation.^[^ ^85^ ^]^ 2) Inflammation and tumor targeting: Platelet membrane adhesion molecules like P‐selectin bind to CD44 receptors on leukocytes, endothelial cells, and tumor cells, serving as therapeutic strategies for inflammation, tumors, and atherosclerosis.^[^ ^108^, ^109^, ^110^, ^111^ ^]^ Additionally, platelet surface integrin αIIbβ3 (glycoprotein IIb/IIIa) mediates platelet‐tumor cell heteroaggregate formation, promoting tumor immune escape.^[^ ^112^ ^]^ 3) Vascular injury targeting: Platelets actively target ischemic vascular endothelium through P‐selectin‐mediated adhesion, facilitating repair processes and modulating local inflammation.^[^ ^113^ ^]^ 4) Immunomodulatory function: Platelet membranes recognize pathogens via Toll‐like receptors and secrete chemokines like C‐X‐C motif chemokine ligand 4 (CXCL4) to recruit leukocytes.^[^ ^114^ ^]^ The primary limitation of platelet‐based BCM‐NHS lies in their tendency to aggregate in vivo, potentially triggering thrombotic events.

Platelets have emerged as versatile platforms for drug delivery and genetic engineering applications due to their unique biological properties. Notably, their natural roles in hemostasis, inflammation, and tumor targeting make them particularly valuable for therapeutic development. In the context of atherosclerosis, Zhang et al. made significant progress by designing platelet membrane‐coated black phosphorus nanosheets (PBP@siR@PM) to deliver CaMKIIγ‐silencing siRNA into macrophages, which remarkably inhibited atherosclerotic progression in high‐fat diet‐fed ApoE⁻^/^⁻ mice.^[^ ^115^ ^]^ Beyond cardiovascular diseases, platelets have shown great promise for inflammatory conditions. A prime example is the work by Feng et al., who developed peptide nanofiber NPs (NLMP2) with hydrophobic surfaces, rough textures, and unique secondary structures. What makes this approach special is their ability to rapidly (<5 s) and efficiently hitchhike on both resting and activated platelets, enabling targeted delivery to pulmonary inflammatory regions through non‐covalent attachment.^[^ ^39^ ^]^ For cancer applications, Dai et al. took advantage of this by constructing platelet membrane‐coated AIE luminogen nanoparticles (Plt‐M@P) for tumor‐targeted photodynamic therapy using PLGA/PF3‐PPh3 complexes.^[^ ^116^ ^]^ Similarly, Yu et al. further engineered platelet‐mimicking PLGA nanoparticles that replicate natural platelet size and surface properties, achieving dual small‐molecule/protein tumor targeting.^[^ ^117^ ^]^ For thrombotic disorders, Sun et al. adopted an innovative approach by developing discoid platelet membrane nanostructures (PLT‐NDs) that not only neutralize pathological antibodies but also prevent platelet depletion while maintaining crucial hemostatic function.^[^ ^113^ ^]^ Collectively, these studies demonstrate how platelet‐inspired drug delivery systems can be precisely tailored for diverse disease applications while leveraging platelets' natural biological behaviors.

#### 3.1.5 Other Cellular Carriers‐Based BCM‐NHS

T cells possess specific receptors capable of recognizing and responding to tumor antigens. Through BCM‐NHS, T cells' anti‐tumor capacity and persistence at tumor sites can be enhanced. For T lymphocytes, a PD‐1 antibody‐modified metal‐organic framework (MOF) combined with liposomal NPs (C&H@MOF/PL‐A) binds to PD‐1 antigens on T cell surfaces during circulation, anchoring NPs to T cells. After T cell‐mediated transport into tumor regions, the release of Hb and CAT from NPs alleviates tumor hypoxia, activating CD8^+^ T cell immune responses to treat colorectal cancer.^[^ ^43^ ^]^ Given that dendritic cells are the most prominent antigen‐presenting cells capable of capturing, processing, and presenting tumor‐associated antigens (TAAs) to trigger T cell‐mediated tumor‐specific immunity,^[^ ^73^, ^118^ ^]^ previous studies designed dendritic cell membrane‐coated AIE photosensitizer NPs (DC@AIEdots). These NPs are recognized and carried by T cells into tumor areas, generating ROS under light irradiation to induce breast cancer cell apoptosis and enhance photodynamic immunotherapy efficacy.^[^ ^119^ ^]^

Mesenchymal stem cells (MSCs) have shown promising results in BCM‐NHS. MSCs are stromal cells differentiating into various connective tissues and capable of following inflammatory mediators to infiltrate the tumor microenvironment (TME). Their non‐pathogenic nature and minimal immunogenicity make them ideal carriers with targeted delivery capabilities. Han et al. developed iron‐doped melanin NPs (FM NPs) combined with MSCs to form Janus structures (MSCFM), where NPs are linked to one side of the MSC surface while preserving natural functionality on the other. These structures migrate to inflammatory sites, suppress Th17 cell proliferation, promote Treg cells, scavenge free radicals, and reduce oxidative stress to alleviate RA pathological progression.^[^ ^55^ ^]^ Leveraging MSCs' enhanced tumor migration, researchers designed NPs (QD‐Ce6) composed of photoluminescent quantum dots (QDs) and photosensitizer chlorin e6 (Ce6). These NPs are phagocytized by MSCs, transported to tumor regions, and generate singlet oxygen via quantum dot energy transfer under external light stimulation to disrupt cancer cell structures.^[^ ^72^ ^]^

### 3.2 Non‐Cellular Biological Carriers

#### 3.2.1 Albumin

Albumin “hitchhiking” strategy has emerged as an effective strategy to enhance various therapeutic agents. Albumin plays vital roles in maintaining intravascular colloidal osmotic pressure, neutralizing toxins, and transporting drugs.^[^ ^67^ ^]^ The advantages of albumin as a nanocarrier include^[^ ^2^ ^]^: 1) Abundant availability: Albumin constitutes the most abundant protein in blood, accounting for ∼60% of all blood proteins; 2) Biosafety: As a highly water‐soluble globular protein, albumin exhibits biocompatibility, biodegradability, non‐immunogenicity, and clinical safety; 3) Rich binding sites: Albumin's unique pocket structure with chemical configurations and conformations allows interactions with numerous drugs, potentially protecting them from elimination and metabolism in vivo; 4) Extended half‐life: With a 19‐day half‐life and renal clearance evasion, albumin prolongs the circulation time of nanomedicines; 5) Tumor cell uptake: Albumin primarily relies on receptor‐mediated transcytosis via gp60 receptors and secreted protein acidic and rich in cysteine (SPARC) pathways for tumor internalization, enabling active targeting without external ligands. Leveraging these merits, albumin‐bound nanodrugs like NPs albumin‐bound paclitaxel (ABRAXANE ABI‐007) with higher response rates in squamous NSCLC,^[^ ^2^, ^120^ ^]^ and ABI‐008 (Nab‐docetaxel) for metastatic breast cancer and hormone‐refractory prostate cancer,^[^ ^2^ ^]^ are widely used clinically. Albumin‐NPs conjugation predominantly involves surface attachment through covalent or non‐covalent binding (Table S5, Supporting Information).

Current albumin “hitchhiking” strategies primarily target tumor treatment.^[^ ^121^, ^122^ ^]^ Xu et al. designed an NIR‐II fluorescent probe IR1080 that activates fluorescence upon albumin binding. The probe's high‐affinity interaction with SPARC overexpressed in tumors amplifies fluorescence signals, enabling precise detection of <2 mm micro‐metastases for surgical navigation.^[^ ^51^ ^]^ Addressing KRAS mutation‐driven metabolic addiction (enhanced glycolysis, glutaminolysis, and macropinocytosis) in PDAC, Huang et al. developed a “Trojan horse” nanomaterial (Nutri‐hijacker) using albumin as a drug carrier. This system enters KRAS‐mutant cancer cells via macropinocytosis, employing biguanides and flavonoids to inhibit glycolysis/glutaminolysis and reprogram tumor metabolism.^[^ ^53^ ^]^ Fu et al. assembled highly cytotoxic mertansine (DM1) and Evans blue (EB) into NPs (EB‐ss‐DM1), where EB covalently binds albumin for tumor delivery and DM1 release to suppress tumor growth.^[^ ^52^ ^]^ However, challenges remain: 1) Albumin corona formation: Albumin can form a “protein corona” on NPs surfaces, altering delivery and targeting properties^[^ ^2^ ^]^; 2) Functional alterations of albumin: potential functional alterations of albumin by loaded NPs; 3) NPs release concerns: Failure to release drugs from albumin may induce toxicity.

#### 3.2.2 Bacteria

Bacteria, particularly anaerobic bacteria, can actively target tumors through hypoxia and TME tropism, and have been widely employed as drug carriers for tumor immunotherapy. Simultaneously, bacterial vectors exhibit immunostimulatory and oncolytic effects, synergistically enhancing the anti‐tumor efficacy of nanomedicine. Thus, utilizing bacteria as nanomedicine carriers can partially overcome the limitations of anti‐tumor nanomedicine. Key bacterial types involved include: probiotic‐active Escherichia coli, genetically engineered attenuated Salmonella typhimurium, T cell activity‐enhancing Listeria monocytogenes, photosynthetic bacteria with photothermal conversion capabilities, and magnetic bacteria.^[^ ^123^, ^124^, ^125^, ^126^ ^]^ The construction mechanisms of bacteria‐nanodrug complexes primarily involve surface binding and encapsulation^[^ ^127^, ^128^, ^129^, ^130^ ^]^ (Table S6, Supporting Information).

Current bacterial‐based BCM‐NHS predominantly focus on tumor applications. For tumor imaging, Zhang et al. utilized probiotics to transport ultrasmall hafnium (Hf) NPs (1–2 nm) to the gastrointestinal tract for imaging.^[^ ^131^ ^]^ In tumor therapy, Zhang et al. combined functionalized gold@cerium‐zinc composite core‐shell NPs (AZC‐A) with *Escherichia coli* (*E. coli*) to engineer bacteria (AZCE), which were then integrated with microneedle carriers to create AZCE‐MN. When implanted into triple‐negative breast cancer (TNBC) tumors, *E. coli*’s intrinsic properties enabled AZCE penetration through the extracellular matrix and basement membrane, effectively delivering AZC to tumor regions rich in cancer stem cells (CSCs). AZC released Zn^2^⁺ in the TME to induce mitochondrial damage, while Au nanorods generated photothermal effects to ablate TNBC cells and CSCs.^[^ ^56^ ^]^ Zhang et al. developed a novel mRNA vaccine delivery nanosystem (PSB@Nb1.33C/mRNA) using photosynthetic bacteria (PSB), leveraging their light‐driven and hypoxia‐driven properties to efficiently deliver tumor‐associated antigen mRNA (WT1 mRNA) to core tumor regions. Photothermal effects from PSB and novel 2D material (Nb1.33C) generated heat to kill tumor cells and amplify immune responses.^[^ ^132^ ^]^

Current challenges in bacterial‐based BCM‐NHS include^[^ ^126^ ^]^: 1) Biosafety: Immunocompromised cancer patients risk clinical infections and systemic inflammation from exogenous bacteria.^[^ ^133^ ^]^ Safety improvements require strategies like probiotic‐based delivery^[^ ^131^, ^134^ ^]^ or bacterial derivatives with similar immunogenicity but higher safety.^[^ ^21^, ^135^ ^]^ 2) Dosage optimization: Bacterial biological effects correlate closely with administered doses, yet research on their in vivo behavior and therapeutic mechanisms in tumors remains insufficient. 3) Post‐treatment clearance: Antibiotics commonly used to eliminate bacterial carriers post‐therapy pose toxicity and resistance risks. 4) Off‐target effects: Bacterial colonization in non‐target tissues may trigger inflammation, infections, or functional disruptions. 5) Immune clearance: Rapid host immune recognition and elimination of bacteria before tumor targeting reduces drug delivery efficiency. In summary, bacteria‐mediated tumor therapy still faces significant clinical challenges.

## 4 Physical Properties and Release Mechanisms

### 4.1 Physical Properties of NPs in “Hitchhiking” Strategy

#### 4.1.1 Surface Charge (Cationic vs Anionic Particles)

Previous studies have shown that compared to anionic NPs, cationic NPs exhibit stronger binding to cells in vivo, making them more likely to evade immune clearance. The primary reason lies in the negative charge on the surface of in vivo cells, which generates strong electrostatic attraction with cationic NPs. For example, Zheng et al. designed two types of NPs: anionic NPs represented by PLGA‐encapsulated ivermectin NPs (IVM‐PNPs), and cationic NPs represented by chitosan‐coated ivermectin NPs (IVM‐CSPNPs). The cationic NPs demonstrated higher non‐covalent binding efficiency with red blood cells than their anionic counterparts, resulting in prolonged in vivo retention and enhanced evasion of immune clearance.^[^ ^36^ ^]^ Similarly, due to the inherent negative charge on dendritic cell membranes, dendritic cells exhibit improved uptake of positively charged NPs.^[^ ^136^ ^]^

#### 4.1.2 Size, Shape, Rigidity, and Surface Ligands

The shape and size of NPs significantly influence their behaviors, primarily manifested in the following aspects: 1) Marginalization and adhesion efficiency: Non‐spherical particles exhibit higher marginalization and adhesion efficiency due to rotational motion and a larger effective contact area. For instance, oblate particles aligned parallel to the vessel wall experience minimal fluid resistance and possess a significantly greater adhesion area than spherical particles, thereby enhancing stability.^[^ ^137^, ^138^, ^139^ ^]^ 2) Encapsulation and uptake rates: Nanorods more readily trigger caveolae‐mediated endocytosis, whereas the uptake of nanospheres is typically clathrin‐mediated.^[^ ^138^ ^]^ NPs with a diameter of approximately 50 nm demonstrate optimal cellular uptake and intracellular delivery, while excessively small NPs (<30 nm) may fail to drive membrane wrapping or activate endocytic processes.^[^ ^140^ ^]^ 3) Hepatic and renal clearance: The kidneys and liver are the primary organs for blood filtration and clearance.^[^ ^141^ ^]^ NPs of varying sizes are eliminated via distinct mechanisms and exhibit preferential distribution across organs. NPs smaller than 5–8 nm are rapidly cleared by glomerular filtration units in the kidneys, while medium‐sized NPs (20–150 nm) tend to be cleared by the RES in the liver. NPs larger than 200 nm show greater resistance to RES clearance and accumulate in the liver, spleen, and lungs,^[^ ^14^, ^142^, ^143^, ^144^ ^]^ but carry a high risk of attracting component 3 (C3) to their protein corona and subsequently triggering immune responses.^[^ ^145^ ^]^

NPs stiffness also impacts the encapsulation efficacy by carrier cells, with rigid particles requiring less energy for endocytosis and being more readily internalized.^[^ ^140^ ^]^ Particle elasticity (rigidity vs deformability) significantly influences marginalization and adhesion: rigid particles exhibit higher marginalization and adhesion under high shear stress, while deformable particles show superior performance under low shear stress. The density and quantity of surface ligands critically affect adhesion; NPs functionalized with biomolecules (e.g., biotin) are more readily adsorbed and retained by vascular walls in blood flow.^[^ ^138^ ^]^ Multiple factors collectively contribute to the low efficiency of systemically administered NPs in brain delivery, underscoring the importance of tailoring NPs design to prolong circulation time and minimize nonspecific distribution.^[^ ^146^ ^]^

### 4.2 Release Mechanism for NPs in “Hitchhiking” Strategy

NPs release primarily relies on two mechanisms: stimulation from the pathological microenvironment and external stimuli. Pathological microenvironmental triggers include pH levels, redox conditions, enzyme concentrations, temperature variations, and NETs release. External stimuli encompass ultrasound, temperature and phototherapy, magnetic fields, and other physical triggers. These dual mechanisms enable precise drug release.^[^ ^74^, ^140^, ^147^, ^148^ ^]^ The specific mechanisms are as detailed below:

#### 4.2.1 Release Mechanism with the Intracellular Stimuli

##### pH‐Responsive Release

The extracellular pH in inflammatory and tumor regions is slightly acidic (6.5–7.0), while the intracellular pH is slightly higher than that of normal tissues.^[^ ^149^ ^]^ Certain NPs utilize pH‐responsive polymers or enzyme‐responsive materials to achieve drug release within the tumor microenvironment. This microenvironment‐responsive release mechanism enhances therapeutic efficacy and reduces toxicity to normal tissues. For example, Sun et al. non‐covalently attached pH‐responsive cationic NPs loaded with simvastatin (SIM‐PEI‐PPNPs) to RBC surfaces, creating a novel drug delivery system (RBC@SIM‐PEI‐PPNPs) that transports simvastatin to pneumonic regions, where the acidic environment triggers its release for anti‐inflammatory effects.^[^ ^35^ ^]^ Similarly, NPs surface‐modified with tailored chitosan (SHIDS) leverage GOx encapsulated within the NPs to catalyze glucose into gluconic acid and hydrogen peroxide (H_2_O_2_) in hyperglycemic environments. The resulting acidic pH induces chitosan swelling, releasing insulin to exert hypoglycemic effects.^[^ ^42^ ^]^ Previous researchers developed an integrated oral microgel system (NPYs) that swells in the neutral or weakly alkaline intestinal environment (pH 6.8–7.4), releasing NPYs and lactic acid bacteria. The released NPYs are absorbed into the bloodstream and transported by macrophages to the kidneys, where they mitigate renal fibrosis by modulating the TGF‐β/Smad signaling pathway.^[^ ^150^ ^]^ Wei et al. designed a dual‐mode biosensor (TDHP) based on PNA/peptide copolymers and a DNA tetrahedron for tumor imaging and urine analysis. Macrophages carrying NPs actively migrate and infiltrate tumor sites, where locally upregulated matrix metalloproteinases (MMPs) cleave 4PD to dissociate TDHP from macrophages, enabling its release.^[^ ^38^ ^]^

##### Redox‐Responsive Release

Compared to healthy cells, tumor cells exhibit relatively higher oxidative stress. ROS are byproducts of aerobic metabolism, and glutathione (GSH) levels in tumors are four times higher than in healthy tissues. Based on this redox environment, current intelligent delivery systems primarily incorporate redox‐sensitive stimuli such as disulfide bonds and diselenide bonds. Among these, redox‐responsive delivery systems containing disulfide bonds cleavable by GSH have been extensively studied.^[^ ^149^ ^]^ For example, Fu et al. covalently conjugated the highly cytotoxic drug DM1 (mertansine) with Evans blue (EB) through a responsive disulfide bond, self‐assembling into NPs (EB‐ss‐DM1), where the disulfide bond breaks under the reductive environment of tumor cells (e.g., in the presence of glutathione) to achieve controlled drug release.^[^ ^52^ ^]^ Li et al. utilized β‐cyclodextrin (β‐CD)‐modified RBCs as hosts and Fc‐modified curcumin‐loaded liposomes as guests, attaching NPs to RBCs via host–guest interactions in vitro. Upon reaching inflammatory regions under ROS stimulation, the β‐CD‐Fc host–guest pairs dissociate, causing liposome detachment from RBCs for targeted curcumin release.^[^ ^30^ ^]^ Similarly, previous researchers designed Fc‐modified copper sulfide NPs (SH‐Fc) that hitchhike on macrophages to tumor sites. The oxidation of Fc dissociates the β‐cyclodextrin‐Fc host–guest pairs, triggering disassembly of CuS aggregates into smaller CuS NPs for deep tissue penetration and enhanced photothermal therapy.^[^ ^70^ ^]^ In atherosclerosis research, scientists developed a nanomicelle (Citop‐NMs) based on the amphiphilic polymer DSPP. After being engulfed by monocytes and transported to atherosclerotic plaques, these monocytes differentiate into foam cells, where abundant ROS triggers NPs release of citral and sulfo‐captopril to suppress inflammation and promote anti‐inflammatory cytokine secretion.^[^ ^71^ ^]^

##### Enzyme‐Responsive Release

Enzymes are widely present in tissues and organs to maintain normal human physiological functions. The tumor microenvironment exhibits abnormal expression of enzymes such as matrix metalloproteinases, cathepsins, phospholipases, and redox enzymes, and the development of enzyme‐responsive NPs for tumor microenvironment‐targeted therapy represents another effective strategy for intelligent NPs to deliver payloads to desired targets.^[^ ^149^ ^]^ Previous researchers designed an oral NPs (βGlus‐ZnD) modified with βGlus that accumulates in tumor regions via macrophage homing effects. Under stimulation by lysosomal enzymes in activated macrophages, the chemotherapeutic drug doxorubicin (DOX) is released from the NPs, stimulating macrophage differentiation into M1‐like phenotypes, recruiting effector T cells, and ultimately inducing tumor cell apoptosis.^[^ ^68^ ^]^

##### Thermally Responsive Release

E‐selectin‐modified thermosensitive nanomicelles also deliver doxorubicin and A2A adenosine receptor antagonist SCH 58 261 to tumor regions through leukocyte binding, where heat radiation induces structural disintegration of NPs to release drugs that inhibit tumor growth and metastasis.^[^ ^44^ ^]^ Previous researchers designed bacterial outer membrane vesicles (OMVs) encapsulating photothermal converters (PBIBDF‐BT), transported by neutrophils to tumor areas, where exogenous thermal stimulation triggers nanomaterial structural collapse to release loaded cisplatin for antitumor effects.^[^ ^82^ ^]^

##### Release via NETs

Neutrophils can form NETs by expelling intracellular contents, including neutrophil DNA and associated proteolytic enzymes, which facilitate cargo escape from cellular barriers. For example, drug‐loaded liposome NPs (n‐DOCPs) are taken up by neutrophils and traverse the alveolar‐capillary barrier, releasing loaded dexamethasone and antibiotics through NETs in pneumonia‐affected areas to exert anti‐inflammatory effects.^[^ ^63^ ^]^ Similarly, SA‐modified platelet membrane‐coated and deoxyribonuclease I (DNase I)‐loaded biomimetic nanozymes (D@HPB@SPM NPs) cross the blood–brain barrier via neutrophil “hitchhiking” into damaged brain parenchyma, where NETs generation releases D@HPB@SPM NPs. These NPs not only alleviate oxidative stress injury by efficiently scavenging ROS but also reduce neutrophil‐induced reperfusion injury by degrading NETs, akin to “burning bridges”.^[^ ^62^ ^]^

#### 4.2.2 Release Mechanism with the Extrinsic Stimuli

Phototherapy refers to the spatial and temporal control of drug release through light irradiation, with light sources including visible light, ultrasound, or NIR, where key parameters such as light intensity, wavelength, and exposure time govern the structural and functional modulation of nanomaterials. To apply this technology, photo‐responsive smart NPs require integration of photosensitive components.^[^ ^149^ ^]^ Previous researchers designed dendritic cell membrane‐coated AIE photosensitizer NPs (DC@AIEdots) transported by T cells to breast cancer regions, where the inner AIE molecules selectively accumulate in lipid droplets of tumor cells. Under light irradiation, AIE photosensitizers generate ROS, inducing tumor cell apoptosis via photodynamic therapy (PDT).^[^ ^119^ ^]^ Another study developed NPs (QD‐Ce6) composed of photoluminescent quantum dots (QDs) and photosensitizer c(Ce6), which, after being carried by mesenchymal stem cells to tumor sites, utilize QDs as energy donors under exogenous light stimulation to transfer energy to attached Ce6 via Förster resonance energy transfer (FRET), producing singlet oxygen for combined diagnostic and therapeutic PDT.^[^ ^72^ ^]^

Ultrasound‐responsive smart NPs possess the ability to enhance ultrasound contrast, enabling their use in imaging diagnostics and image‐guided drug delivery. Given the thermal‐responsive properties of ultrasound, it can be employed to construct dual thermal and ultrasound‐responsive smart nanosystems.^[^ ^149^ ^]^

Magnetic‐responsive drug delivery systems typically assemble paramagnetic and superparamagnetic NPs into polymeric scaffolds to enhance tumor accumulation of anticancer drugs under static magnetic fields. Among inorganic nanomaterials, iron oxide NPs (IONPs) have garnered significant interest due to their excellent magnetism, biocompatibility, low toxicity, and biodegradability. Superparamagnetic iron oxide NPs (SPIONs) commonly feature magnetite (Fe_3_O_4_) or maghemite (Fe_2_O_3_) cores with polymer or silica‐conjugated shell structures. Under an external alternating magnetic field (AMF), SPIONs generate substantial heat in the local tumor microenvironment while triggering massive release of loaded drugs.^[^ ^149^ ^]^

## 5 Applications of the “Hitchhiking” Strategy in Various Diseases

Benefiting from the advantages of the BCM‐NHS, numerous NPs have been applied in biomedical fields via this strategy. This section will summarize recent advances in BCM‐NHS used for cancer therapy, ischemic stroke, pneumonia, and diagnostic imaging.

### 5.1 Tumor

Cancer remains a leading cause of death worldwide, characterized by rapid proliferation and high invasiveness.^[^ ^151^, ^152^ ^]^ Nanomedicine demonstrates significant potential in tumor diagnosis and treatment.^[^ ^153^, ^154^ ^]^ The accumulation of conventional nanomedicines at tumor sites largely relies on the EPR effect caused by tumor vascular extravasation.^[^ ^155^ ^]^ Tumor cell surfaces express various specific receptors (e.g., antibodies, peptides, transferrin, folate), and specific ligands can be modified on NPs surfaces, where the specific binding between receptors and their corresponding ligands enables targeted drug delivery in vivo.^[^ ^149^ ^]^ The therapeutic agents of nanomedicines in oncology primarily encompass the following aspects:

#### 5.1.1 Tumor Diagnosis

Non‐invasive diagnostic imaging holds immense promise in advancing early disease detection and treatment, with common techniques for in vivo diagnostic imaging including positron emission tomography (PET), magnetic resonance imaging (MRI), computed tomography (CT), ultrasound imaging, and so on.^[^ ^156^, ^157^, ^158^, ^159^ ^]^ These techniques typically rely on imaging probes such as radionuclides, metal ions, and photosensitizers to visualize targeted structures or pathological changes. In recent years, several innovative probes capable of “hitchhiking” on circulating cells or proteins have been developed and widely utilized. This BCM‐NHS endows these imaging probes with two notable advantages: prolonged half‐life and significantly enhanced targeting capability to diseased tissues.

**Figure 2:**
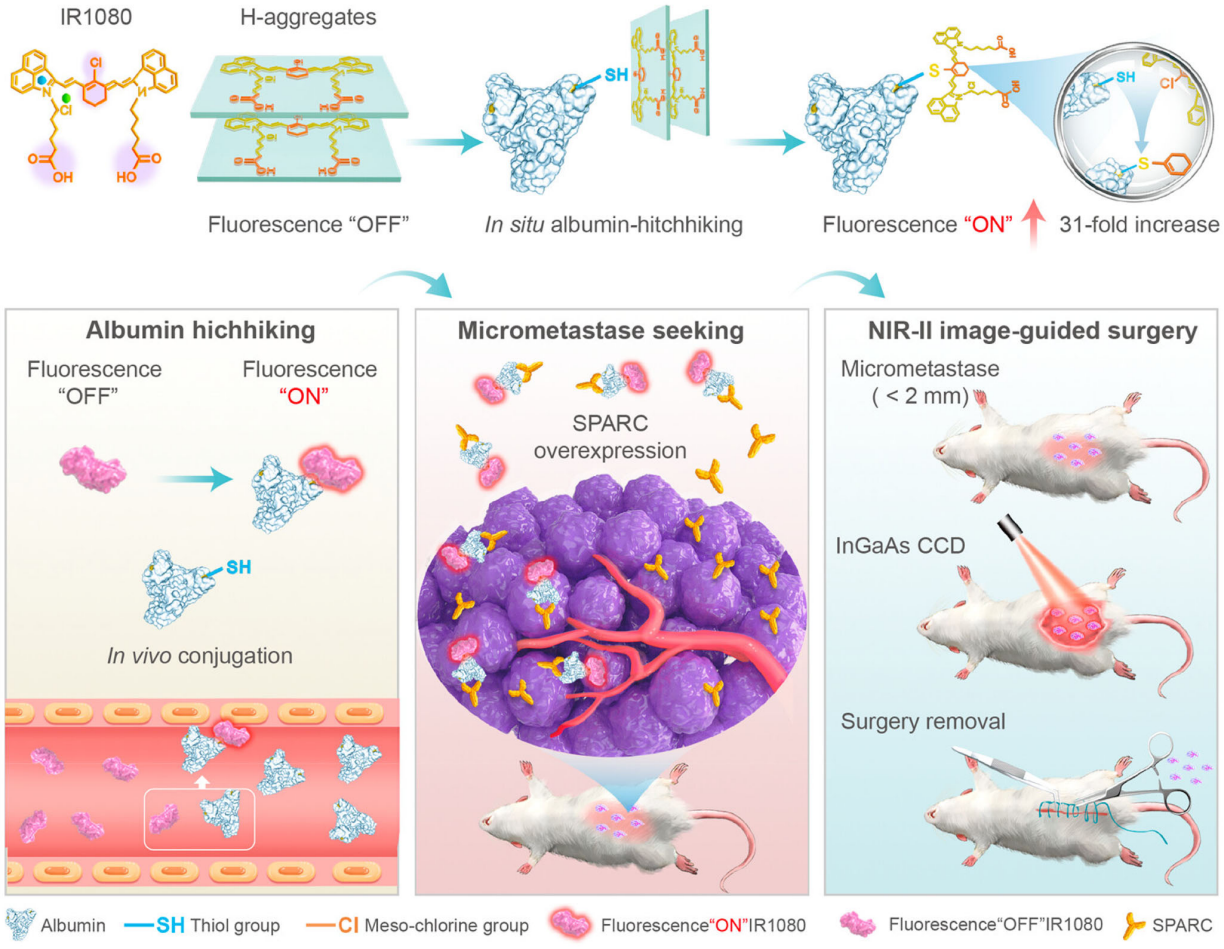
Schematic diagram of a near‐infrared window II (NIR‐II) fluorescent probe, IR1080, exerting chemotherapeutic effects against gliomas. Reproduced with permission.^[^ ^51^ ^]^ Copyright 2023, American Chemical Society.

**Figure 3:**
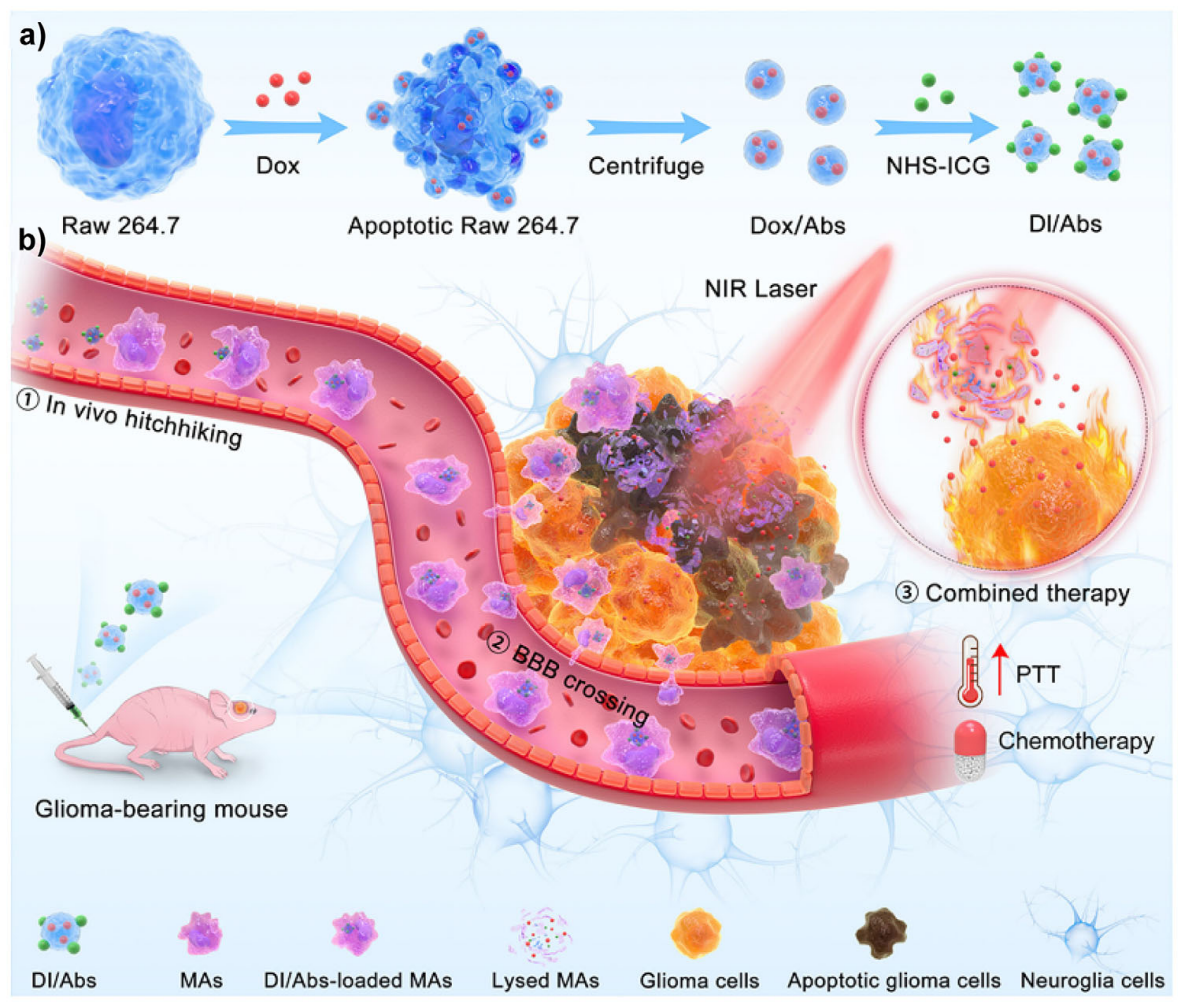
Schematic diagram of macrophage apoptotic body‐encapsulated NPs (DI/Abs) exerting chemotherapeutic effects against gliomas. Reproduced with permission.^[^ ^160^ ^]^ Copyright 2023, Ivyspring International.

**Figure 4:**
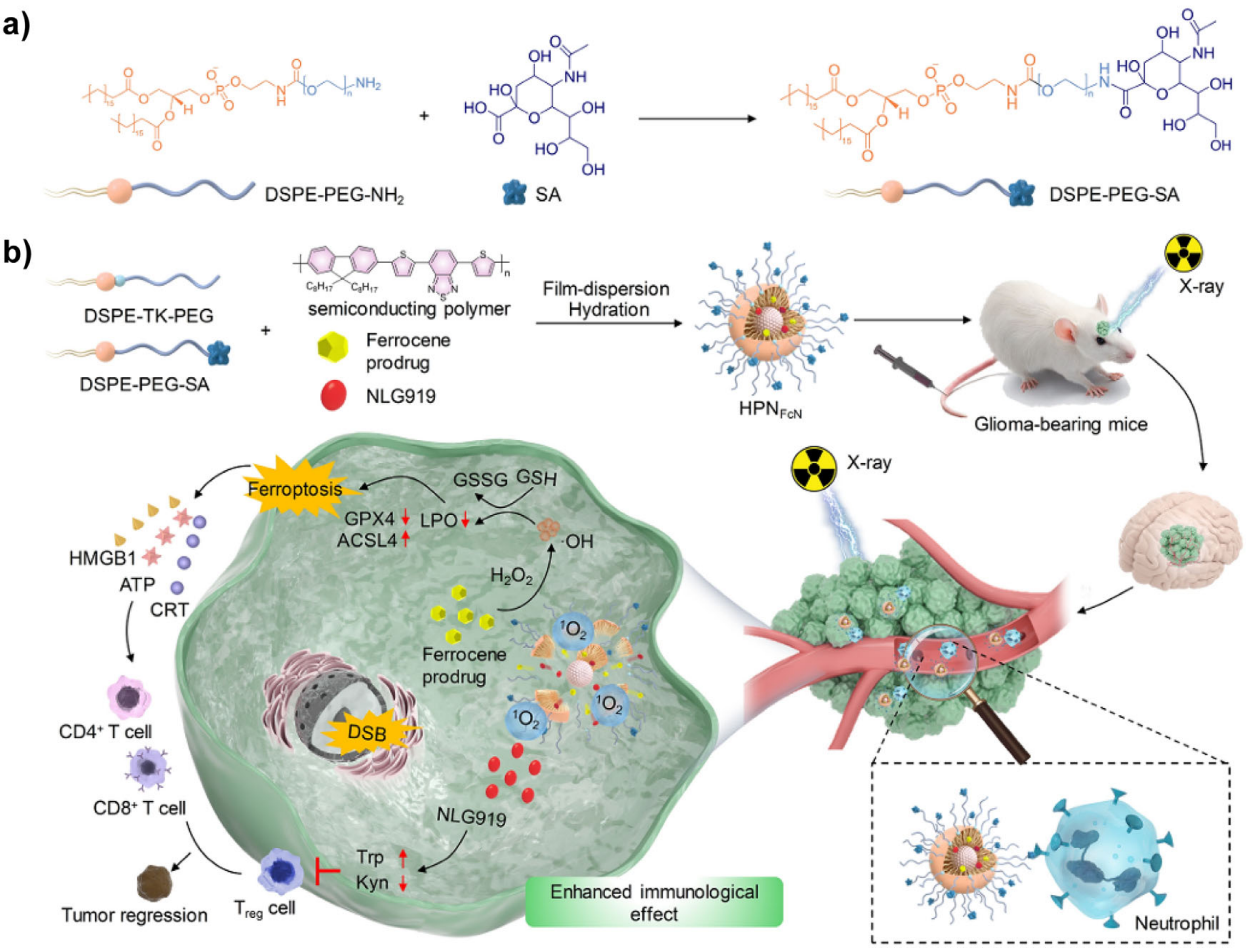
Schematic diagram of X‐ray‐activatable HPNFcN for effective orthotopic glioma therapy. a) Chemical synthesis of DSPE‐PEG‐SA. b) Schematic diagram of the synthesis course of HPNFcN and utilization of HPNFcN for orthotopic glioma therapy via X‐ray mediated activation of ferroptosis and immunization. Reproduced with permission.^[^ ^46^ ^]^ Copyright 2024, Elsevier Ltd.

**Figure 5:**
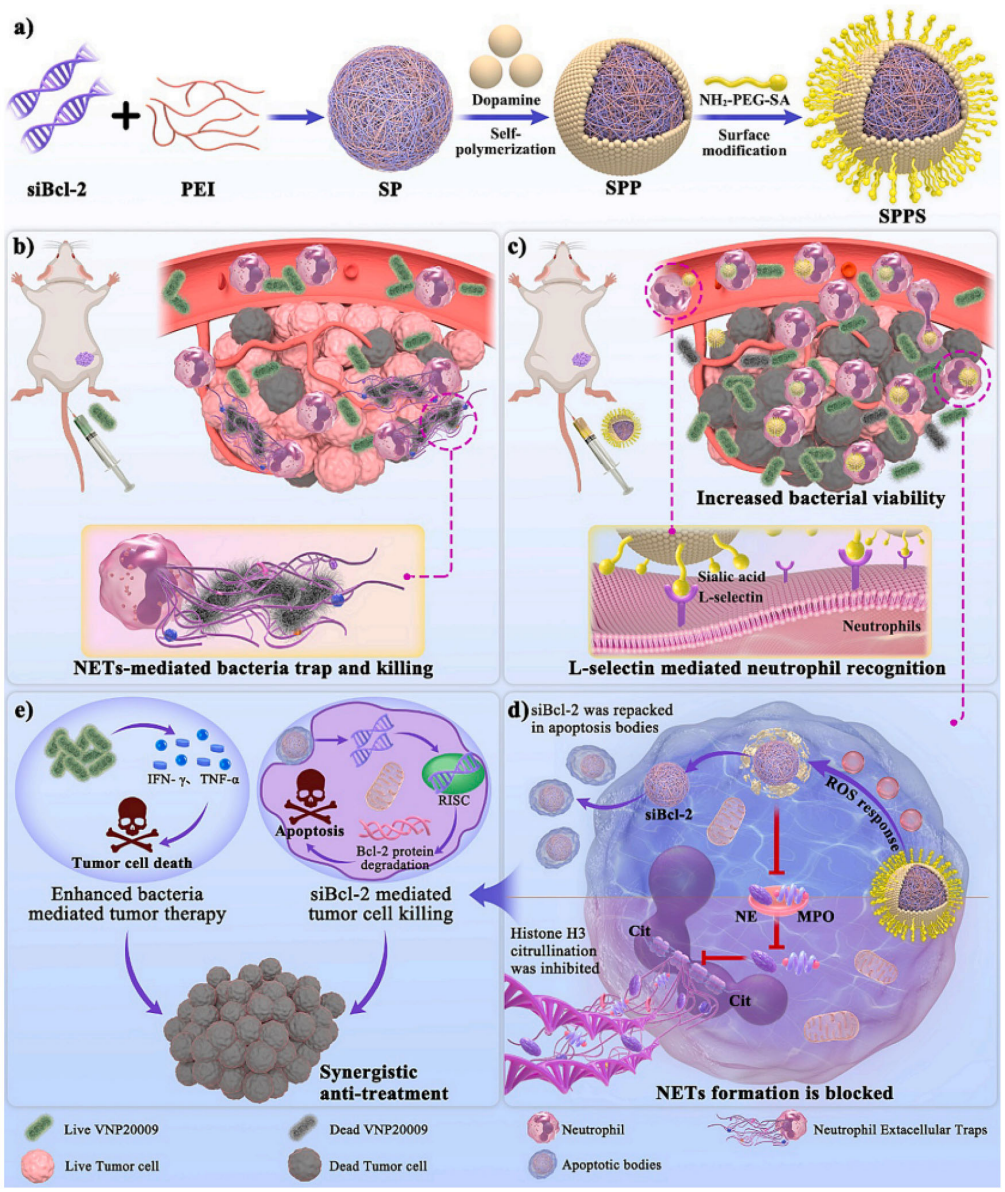
Schematic illustration showing the neutrophils “hitchhiking” nanoparticle boosting bacteria‐mediated tumor therapy via NETosis reprogramming. a) Schematic showing the preparation of SPPS NPs. b) The tumor‐colonized bacteria recruit neutrophils into the tumor, while the formed NETs capture and kill bacteria. c) Neutrophils recognize SPPS NPs and were subsequently recruited into the tumor site. d) Schematic illustration of SPPS‐mediated the shift of neutrophil death mode from NETosis to apoptosis. e) Enhanced bacterial therapy combined with gene therapy for antitumor treatment. Reproduced with permission.^[^ ^61^ ^]^ Copyright 2024, Elsevier Ltd.

**Figure 6:**
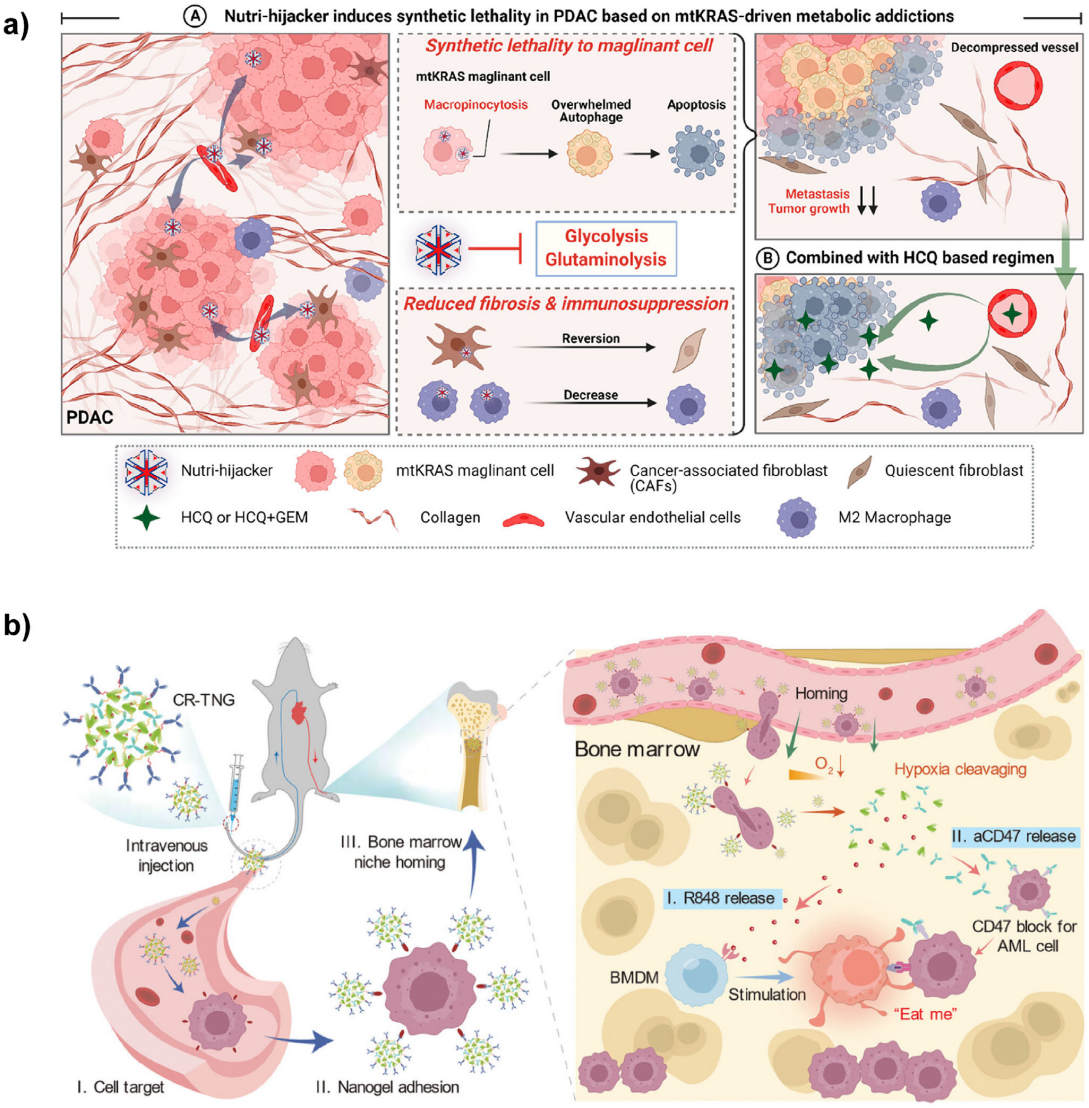
a) Schematic illustration of the phenomenon that Nutri‐hijacker induced synthetic lethality based on mtKRAS‐driven metabolic addiction via inhibiting glycolysis and glutaminolysis after macropinocytosis in mtKRAS malignant cells. Reproduced with permission.^[^ ^53^ ^]^ Copyright 2023, American Chemical Society. b) Schematic illustration of the phenomenon that the potential targeting of aTIM‐3‐modified nanogel CR‐TNG could hijack circulating leukemia cells and home to the bone marrow. Reproduced with permission.^[^ ^50^ ^]^ Copyright 2024, Wiley‐VCH GmbH.

**Figure 7:**
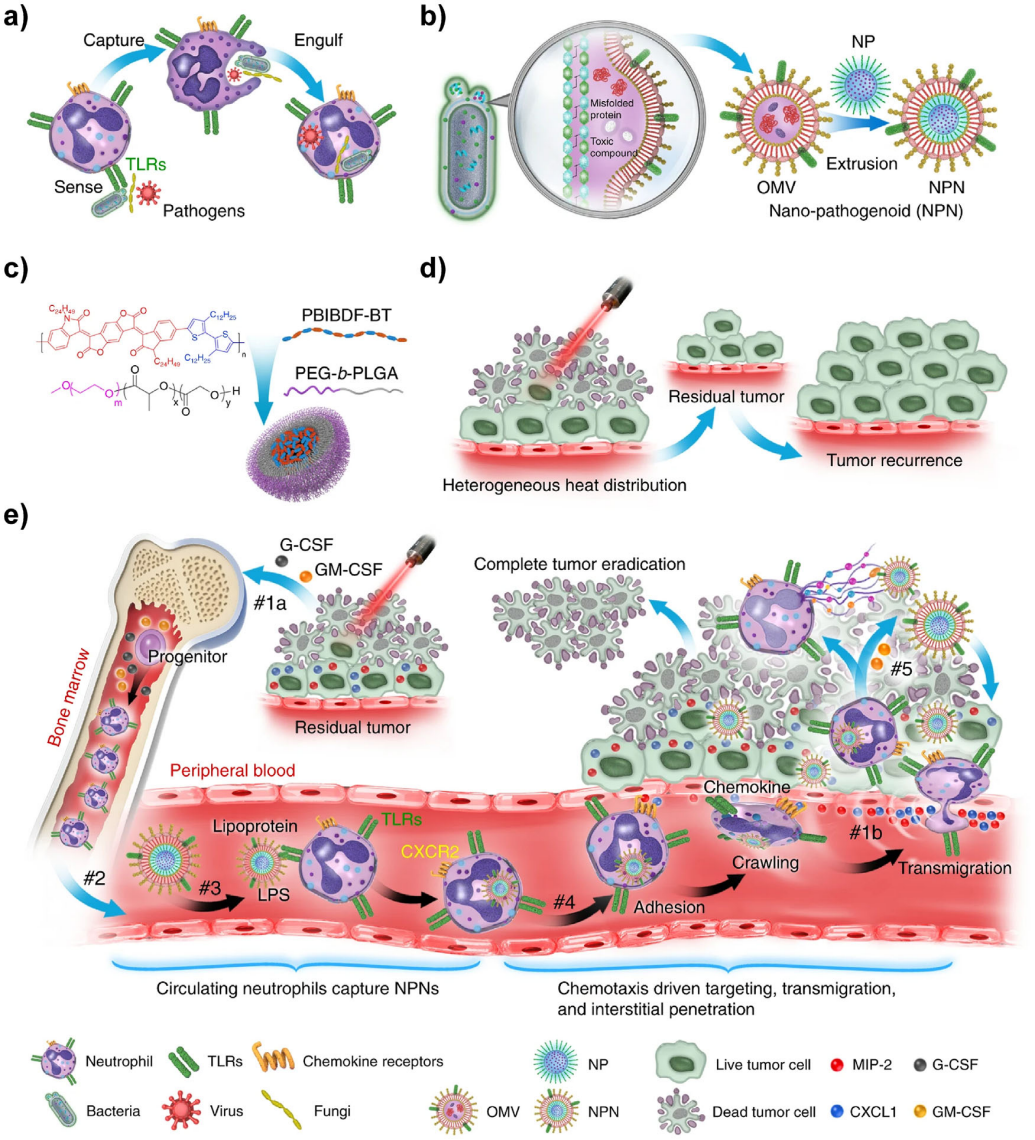
Schematic illustration showing the chemotaxis‐driven delivery of NPNs for complete eradication of tumors post‐phototherapy. a) Neutrophils sense, capture, and engulf pathogens by recognizing the PAMPs with toll‐like receptors (TLRs). b) Preparation of NPNs by coating OMVs on NPs, which inherit PAMPs from the OMVs. c) Preparation of PEG‐b‐PLGA NPs encapsulating PBIBDF‐BT (PBT) as a photothermal transducer. d) The limited penetration of laser light used in PTT causes heterogeneous heat distribution within the tumor tissue and incomplete eradication of tumors, thus leading to tumor recurrence. e) Treatment‐induced cell death created an inflammatory environment of the residual tumor and induced the production of granulocyte colony‐stimulating factor (G‐CSF), granulocyte‐macrophage colony‐stimulating factor (GM‐CSF), and chemokines CXCL1 and MIP‐2. #1a) The released G‐CSF and GM‐CSF increased neutrophil production from bone marrow. #1b) The released CXCL1 and MIP‐2 broadcasted the location of the inflamed tumor. #2) Neutrophils entered the blood circulation and encountered the injected NPNs. #3) Neutrophils sensed NPNs with the recognition of LPS and lipoprotein by TLRs and subsequently engulfed them. #4) Neutrophils laden with NPNs were recruited into the tumor site in response to the chemokine gradient through the following cascade: adhesion, crawling, and transmigration. #5) NPNs were released from neutrophils to kill tumor cells along with the formation of NETs in the inflamed tumor. Reproduced with permission.^[^ ^82^ ^]^ Copyright 2020, Springer Nature.

**Figure 8:**
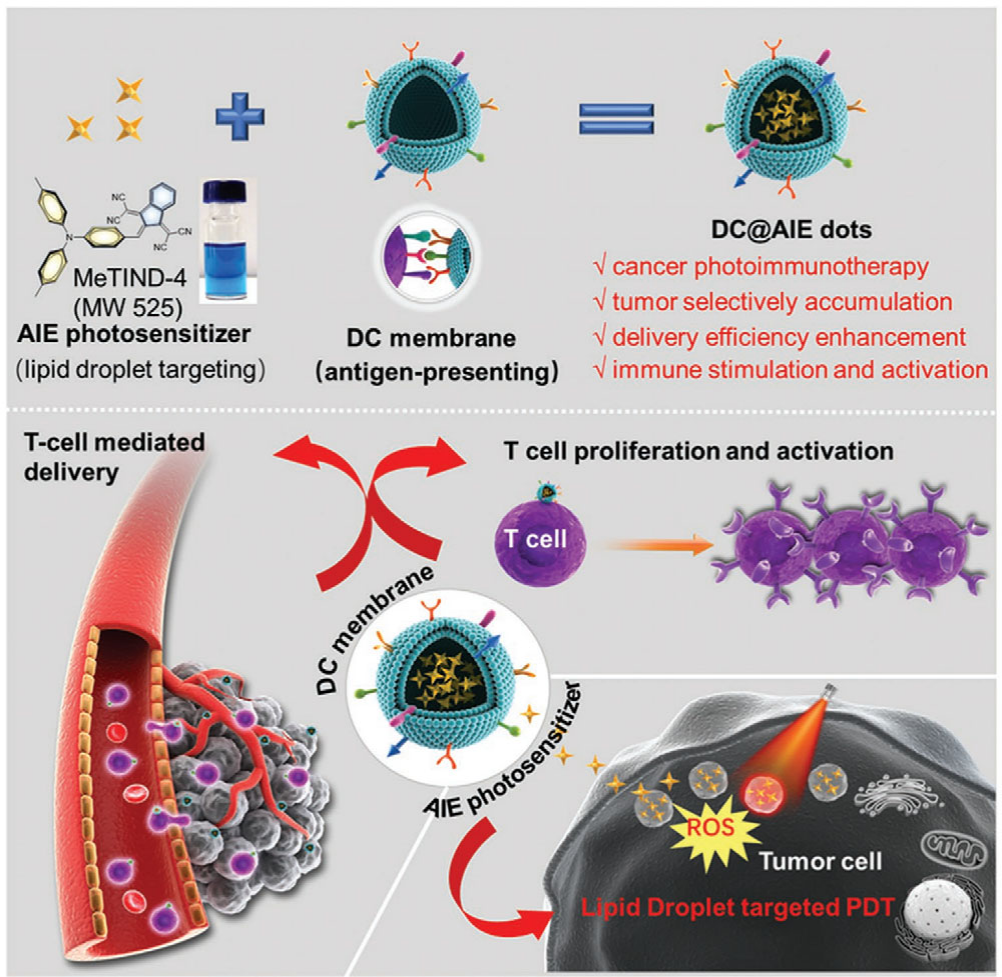
The schematic illustration represents the preparation and assembly of AIE photosensitizers with dendritic cell membrane coating (DC@AIEdots) and in vivo photodynamic immunotherapy. The inner AIE photosensitizers selectively accumulated in tumor cells, and the outer cell membrane facilitated DC@AIEdots to hitchhike on endogenous T cells to enhance the tumor delivery efficiency and stimulate in vivo T cell proliferation and activation to activate the immune system. Reproduced with permission.^[^ ^119^ ^]^ Copyright 2021, Wiley‐VCH GmbH.

**Figure 9:**
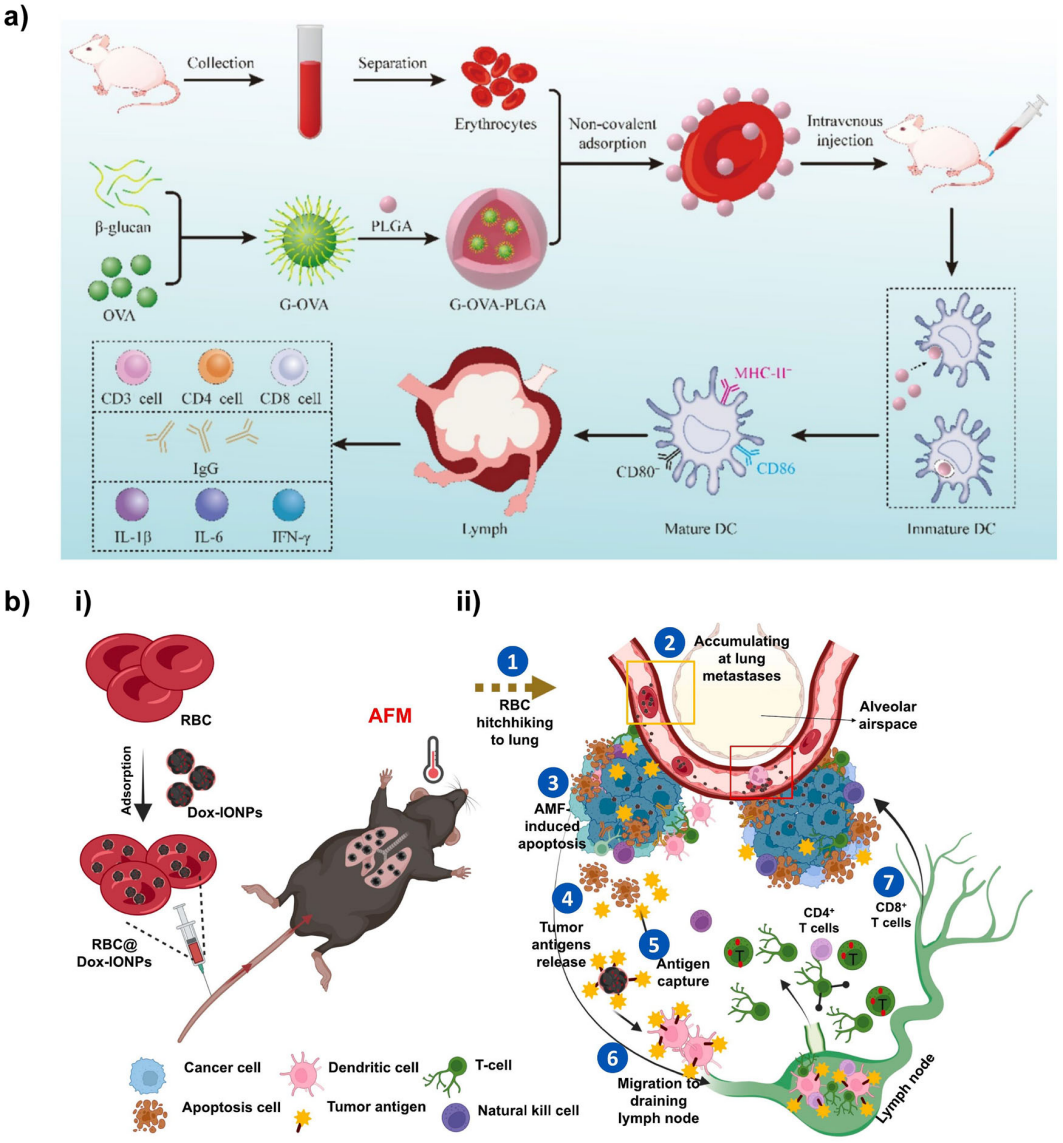
a) Schematic diagram of preparing the G‐OVA‐PLGA@RBC delivery system. Reproduced with permission.^[^ ^34^ ^]^ Copyright 2024, Frontiers. bi) Schematic illustration of RBC‐hitchhiking delivery of MIO to lung metastasis for immune therapy. bii) MIO penetrates into lung metastasis via the endothelial cells and leukocyte uptake at aleveolar airspace, and then, absorbs energy and causes tumor cell apoptosis. The frictional heat on MIOs by an alternating magnetic field (AFM) leads the tumor call apoptosis to release of tumor‐associated antigens, facilitating the recruitment of T cells. Reproduced with permission.^[^ ^29^ ^]^ Copyright 2023, Elsevier Ltd.

**Figure 10:**
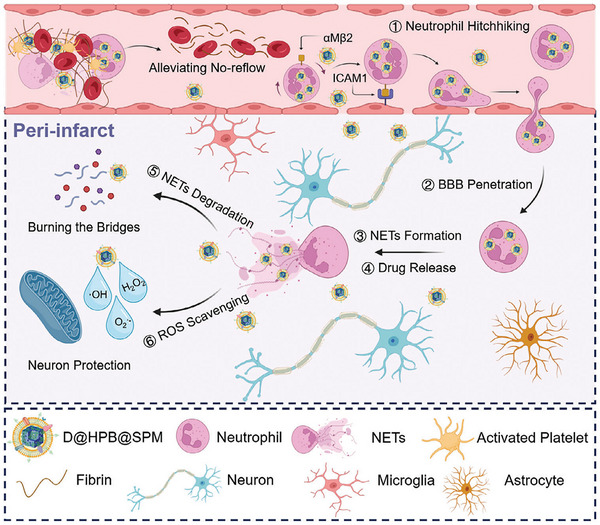
Schematic diagram of the neutrophil “hitchhiking” biomimetic nanozymes mediated ischemic stroke therapy. D@HPB@SPM NPs are specifically endocytosed by neutrophils and hitchhike on neutrophils to reach the cerebral ischemic sites. Subsequently, neutrophils are activated and unleash D@HPB@SPM NPs through the production of NETs. D@HPB@SPM NPs not only mitigate neutrophil‐induced brain damage by degrading NETs in a manner similar to “burning the bridges”, but also relieve oxidative stress injury by efficiently scavenging ROS. Reproduced with permission.^[^ ^181^ ^]^ Copyright 2024, Wiley‐VCH GmbH.

**Figure 11:**
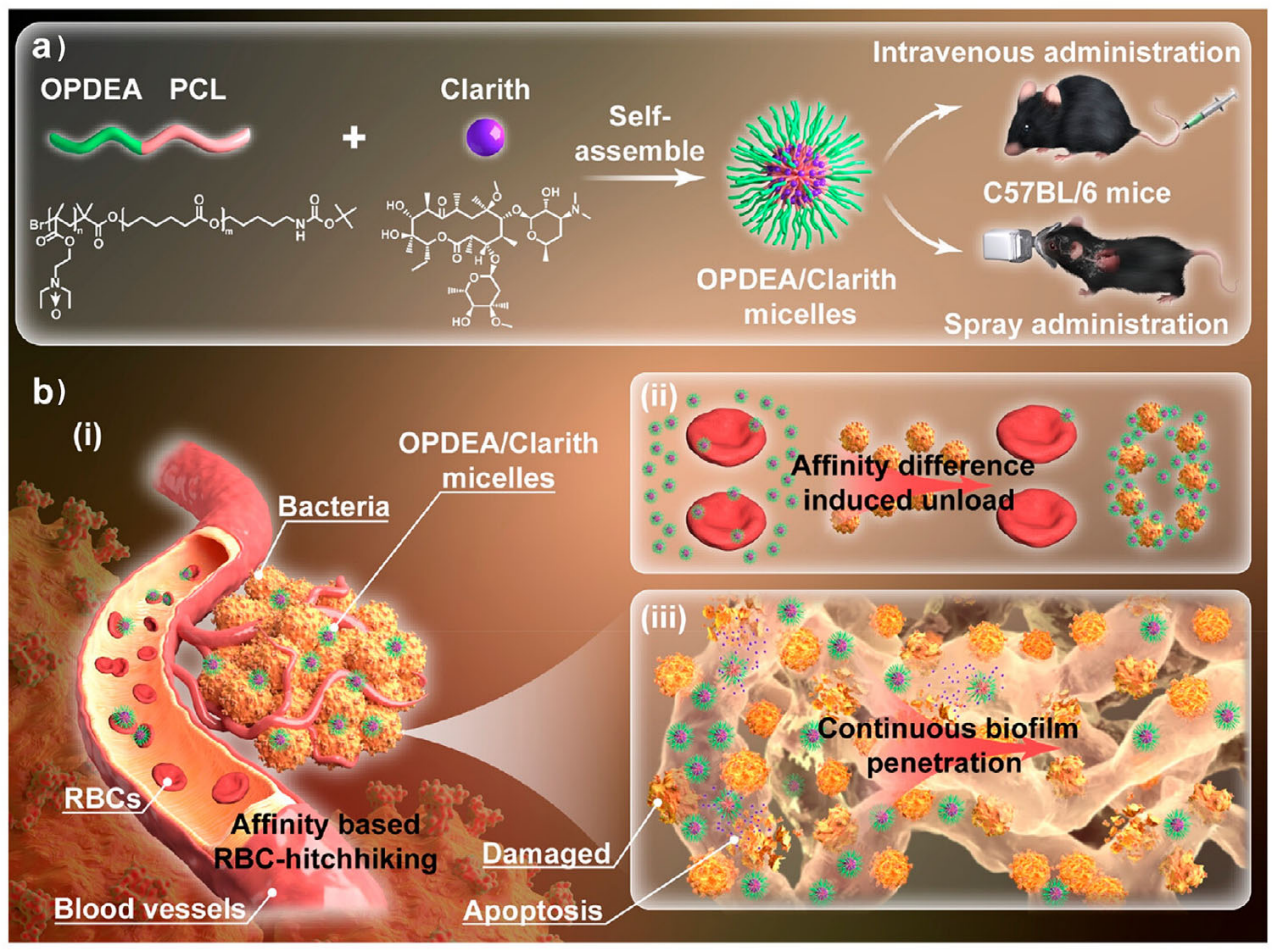
a) Illustration of the self‐assembly of Clarith‐loaded OPDEA−PCL micelles and treatment for the bacterial infection model through intravenous or pulmonary spray administration in C57BL/6 mice. b) Clarith‐loaded OPDEA−PCL micelles i) hitchhike on RBCs in blood circulation, ii) detach from RBCs via the enhanced interaction with the Gram‐positive bacteria cell wall and improve the accumulation, and iii) penetrate the biofilm and boost antibacterial efficacy. Reproduced with permission.^[^ ^105^ ^]^ Copyright 2023, American Chemical Society.

Consequently, specially engineered NPs have been applied for tumor imaging diagnosis and biosensors, achieving high sensitivity and resolution in tumor detection. For instance, MCP1‐modified micellar nanomaterials (MCP1‐Gd micelles) are carried by monocytes to metastatic lymph nodes, where targeted accumulation of gadolinium (Gd) enhances MRI precision for detecting metastatic lymph nodes,^[^ ^41^ ^]^ thereby aiding early lymph node biopsy. Xu et al. designed a near‐infrared window II (NIR‐II) fluorescent probe, IR1080, which forms H‐aggregates in its unbound state with albumin, leaving fluorescence in an “off” state. Upon covalent binding to albumin, the fluorescence switches to an “on” state. The high affinity between overexpressed SPARC in micrometastases and IR1080 further amplifies the fluorescent signal. This targeting capability enables IR1080 to precisely detect micrometastases (<2 mm).^[^ ^51^ ^]^ In previous studies, macrophage apoptotic body‐encapsulated NPs (DI/Abs) were internalized by monocytes/macrophages to cross the blood‐brain barrier into brain tumor tissues. Indocyanine green (ICG), a photothermal agent loaded in the NPs, allows real‐time monitoring of DI/Abs distribution and accumulation in vivo under near‐infrared (NIR) laser irradiation. Its photothermal effect not only destroys tumor cells but also ruptures macrophages to release doxorubicin, exerting chemotherapeutic effects against gliomas^[^ ^160^ ^]^ (**Figures** **2**, **3**, **4**, **5**, **6**, **7**, **8**, **9**, **10**, **11**).

#### 5.1.2 Chemotherapy and Gene Therapy

Through rational design and fabrication of nanomedicine carriers, targeted drug delivery in tumor therapy can be achieved. NPs enable efficient transport of chemotherapeutic agents to tumor tissues via active targeting processes while minimizing damage to healthy tissues.^[^ ^161^ ^]^ For example, Zhu et al. utilized sialic acid (SA)‐modified semiconducting polymer NPs (HPNFcN) hitchhiked by neutrophils to reach glioma regions, releasing ferroptosis inducers and the immunotherapeutic drug NLG919. Under X‐ray assistance, these NPs induced tumor cell ferroptosis and triggered immunogenic cell death.^[^ ^46^ ^]^ In another study, researchers developed bacterial membrane‐doped liposomes (B‐Lipo) loaded with 1‐methyl‐D‐tryptophan (1‐MT) and curcumin (Cur) to form B‐Lipo/1‐MT&Cur NPs. These were efficiently internalized by neutrophils and delivered to brain tumors, eliciting robust antitumor immunostimulatory effects.^[^ ^135^ ^]^ A biomimetic NPs combining arsenic with apoptotic bodies (c) was designed to be transported by macrophages to tumor sites, inducing toxicity or apoptosis in hepatocellular carcinoma cells.^[^ ^162^ ^]^ SA‐modified NPs (SPPS) migrated to tumor regions with neutrophils, where PDA shells inhibited NETs preserving the antitumor activity of attenuated Salmonella strain VNP20009 by reducing neutrophil‐mediated bacterial clearance. Simultaneously, gene therapeutic siBcl‐2 was released to enhance tumor cell apoptosis.^[^ ^61^ ^]^

Multiple genetic mutations and adaptations drive the formation of unique tumor microenvironments (TMEs), often resulting in the upregulation of tumor‐specific biomarkers.^[^ ^163^, ^164^, ^165^ ^]^ Among these, SPARC stands out as a key target—it is highly overexpressed in many malignant tumors but minimally present in normal tissues, making it an ideal candidate for tumor‐specific drug delivery.^[^ ^2^, ^166^, ^167^, ^168^, ^169^ ^]^ Capitalizing on SPARC's affinity for albumin, Chang et al. engineered a nanocomplex (rTCS‐PTN‐ABD/HSA) by noncovalently binding the albumin‐binding domain (ABD) of a fusion protein circulating albumin. In the TME, legumain (a cysteine protease) specifically cleaves the PTN (Pro‐Thr‐Asn) linker in the NPs, releasing trichosanthin (TCS) to inhibit ribosomal protein synthesis in tumor cells, thereby exerting antitumor effects.^[^ ^37^ ^]^ The therapeutic potential of albumin‐mediated delivery is further underscored by the clinical success of Abraxane® (albumin‐bound paclitaxel), an FDA‐approved nanomedicine for breast cancer, non‐small cell lung cancer, and pancreatic adenocarcinoma since 2005.^[^ ^153^ ^]^ Unlike conventional paclitaxel formulations, Abraxane exploits endogenous albumin pathways to enhance tumor accumulation, demonstrating the viability of albumin as a versatile nanocarrier.

Given that KRAS mutations in PDAC drive metabolic dependencies on enhanced glycolysis, glutaminolysis, and macropinocytosis, Huang et al. designed a “Trojan horse” nanomaterial termed Nutri‐hijacker. Utilizing albumin as a drug carrier, it enters KRAS‐mutant malignant cells via macropinocytosis and reprograms tumor metabolism through covalently conjugated biguanides (inhibiting glycolysis) and flavonoids (suppressing glutaminolysis), inducing synthetic lethality while reducing tumor fibrosis and immunosuppression.^[^ ^53^ ^]^

For acute myeloid leukemia (AML) cells expressing CD47 and TIM‐3 antigens, anti‐CD47 antibody (aCD47)‐functionalized protein nanogel particles (CR‐TNG) anchor to leukemia cells by binding CD47 and TIM‐3, blocking CD47‐SIRPα interactions to promote macrophage phagocytosis of AML cells. Concurrently, resiquimod (R848) is released for immunomodulation, achieving therapeutic efficacy against AML.^[^ ^50^ ^]^

#### 5.1.3 Radiosensitization and Photothermal Therapy

On one hand, combining external stimuli such as photothermal therapy (PTT), magnetic hyperthermia therapy (MHT), ultrasound (US), and radiation therapy enhances the therapeutic efficacy of nanosystems.^[^ ^23^ ^]^ On the other hand, A variety of NPs have been developed for the delivery of radionuclides, thereby improving the treatment of tumors.^[^ ^170^ ^]^ For example, the structure of OMV@PBIBDF‐BT NPs primarily involves bacterial outer membrane vesicles (OMVs) encapsulating photothermal converters (PBIBDF‐BT). Through interactions between surface pathogen‐associated molecular patterns (PAMPs) and neutrophil pattern recognition receptors (PRRs), these NPs are transported by neutrophils to tumor regions, releasing cisplatin to synergistically enhance antitumor effects with photothermal therapy.^[^ ^82^ ^]^ In previous studies, gold‐platinum (Au‐Pt) bimetallic nanozyme‐coated bacterial carriers (Au‐Pt@VNP20009, APV) were phagocytized by monocytes/macrophocytes and delivered to tumor sites. The Au‐Pt bimetallic nanozymes efficiently decompose H_2_O_2_ to generate oxygen (O_2_), while the gold (Au) and platinum (Pt) elements enhance X‐ray energy deposition, thereby improving radiotherapy‐induced tumor cell killing.^[^ ^171^ ^]^ Another study constructed bacterial‐mimetic gold NPs (GNPs) via membrane coating and derivatization processes. Phagocytic immune cells transport intracellular GNP aggregates to melanoma tissues through inflammatory tropism. Initial PTT on tumors induces tumor damage, creating positive feedback to recruit more immune cell‐based carriers for enhanced targeting efficiency. Optimized secondary PTT significantly improves antitumor immunotherapy and further amplifies therapeutic outcomes through immune checkpoint blockade.^[^ ^21^ ^]^ Researchers also designed dendritic cell membrane‐coated AIE photosensitizer NPs (DC@AIEdots), which bind to T cells via interactions between membrane surface ligands and corresponding T cell receptors. Transported by T cells to breast cancer regions, the inner AIE molecules selectively accumulate in lipid droplets of tumor cells. Under light irradiation, AIE photosensitizers generate ROS, inducing tumor cell apoptosis via PDT.^[^ ^119^ ^]^

#### 5.1.4 Immunotherapy

Immunotherapy is the most rapidly growing drug class and have a major impact on oncology and on human health.^[^ ^172^, ^173^ ^]^ On the one hand, immune cells can stimulate systemic anti‐tumor immunity and bypass substantial barriers related to tumor targeting. On the other hand, immune cells demonstrate remarkable ability to penetrate the body's natural barriers. Nanomaterials are widely used in tumor immunotherapy.^[^ ^174^ ^]^ As we know, antigen presentation plays an important role in tumor treatment.^[^ ^175^, ^176^, ^177^ ^]^ Therefore, previous studies have developed NPs to specifically modulate immune cells, thereby obtaining potent anti‐tumor activity.^[^ ^178^ ^]^ Folate (FA) functionalization on liposome surfaces can attract natural IgM for non‐covalent binding. A previous study designed a folate‐modified liposomal NPs (FA‐sLip/OVA/MPLA) that attaches to blood IgM for “hitchhiking” to the spleen, delivering the co‐loaded tumor model antigen OVA to MZB cells in the spleen, while releasing the TLR4 agonist (MPLA) immunoadjuvant to induce antigen‐specific humoral immunity and cytotoxic T lymphocyte responses.^[^ ^32^ ^]^ Li et al. developed a β‐glucan‐ovalbumin composite NPs based on PLGA encapsulation (G‐OVA‐PLGA), which was non‐covalently loaded onto red blood cell surfaces to construct a G‐OVA‐PLGA@RBC system. This system releases β‐glucan to promote macrophage polarization towards M1‐type activation and releases ovalbumin (OVA) as a model antigen, which is processed by antigen‐presenting cells to activate T cells.^[^ ^34^ ^]^ Liang et al. designed a MIO‐based doxorubicin‐loaded NPs (Dox‐IONPs) transported by red blood cells to lung tissue. AMF‐mediated magnetic hyperthermia, MIO also acts as an antigen‐capture agent, delivering these antigens to lymph nodes through dendritic cells, thereby improving survival rates and immune responses in mice bearing lung tumor metastases.^[^ ^29^ ^]^

### 5.2 Ischemic Stroke

Ischemic stroke is one of the leading causes of death and disability worldwide, posing significant challenges in both prevention and treatment. Ischemic stroke is caused by blockage of blood vessels supplying the brain, potentially leading to severe neurological deficits and long‐term disability.^[^ ^179^, ^180^ ^]^ Various nanomaterials have been developed for ischemic stroke treatment. For example, biomimetic nanozymes (D@HPB@SPM NPs) are transported by neutrophils to ischemic stroke lesions, clearing ROS and degrading NETs, thereby alleviating neutrophil‐induced reperfusion injury.^[^ ^181^ ^]^ Previous studies utilized PLGA NPs (T‐TMP) targeted to ischemic stroke lesions via neutrophil chemotaxis, releasing the NPs‐loaded ligustrazine to reduce reperfusion damage.^[^ ^47^ ^]^ Trac et al. synthesized integrated cascade enzymes (h‐ANEs) through a one‐pot method, leveraging neutrophil inflammatory tropism to cross the blood‐brain barrier. These enzymes release antioxidant enzymes CAT and SOD1 to eliminate ROS via cascade reactions, while releasing selenium (Se) to enhance GPX4 expression, inhibiting neuronal ferroptosis and ultimately treating I/R‐induced brain injury.^[^ ^48^ ^]^ In previous research, the neuroprotective agent pioglitazone (PGZ) was encapsulated into OMV, forming OMV@PGZ NPs, which are delivered by neutrophils to ischemic brain injury regions. OMV@PGZ nanomaterials simultaneously suppress nucleotide‐binding oligomerization‐like receptor protein 3 (NLRP3) inflammasome activation and ferroptosis, thereby reducing reperfusion injury and exerting neuroprotective effects.^[^ ^182^ ^]^

### 5.3 Pneumonia

Pneumonia is a major global public health concern,^[^ ^183^, ^184^ ^]^ causing great mortality and morbidity and high costs worldwide.^[^ ^185^, ^186^ ^]^ In the forefront of contemporary medical research, BCM‐NHS for pneumonia treatment has emerged as a highly prominent research focus. The following elaborates their applications in pneumonia treatment from various perspectives.

NPs demonstrate immense potential in targeted antibiotic delivery for pneumonia treatment. Leveraging their unique physicochemical properties, NPs can interact with cells in biological systems to achieve precise drug delivery. For instance, Yu et al. constructed OPDEA‐PS micelles based on zwitterionic polymers, where OPDEA binds to red blood cell membranes via phospholipid‐related affinity and transfers to Gram‐positive bacteria due to interactions with bacterial cell walls that are nearly an order of magnitude stronger. Consequently, these micelles can hitchhike on red blood cells, ultimately releasing at infection sites to deeply penetrate biofilms and discharge clarithromycin for antibacterial activity.^[^ ^105^ ^]^ Zheng et al. designed ivermectin‐loaded NPs encapsulated in PLGA (IVM‐PNPs) and chitosan‐coated ivermectin NPs (IVM‐CSPNPs), which enhance antifungal drug accumulation in lung tissue through red blood cell‐targeted transportation, significantly alleviating acute lung injury progression.^[^ ^36^ ^]^

Beyond antibiotics, anti‐inflammatory drugs also play critical roles in pneumonia treatment via NPs‐mediated targeted delivery. Li et al. developed Fc‐modified curcumin‐loaded liposomes that hitchhike on red blood cells to inflammatory regions. Upon ROS stimulation, the liposomes detach from red blood cells, enabling curcumin's specific release to effectively mitigate acute pneumonia symptoms.^[^ ^30^ ^]^ PDA NPs with surface CPPC modification are transported by neutrophils to acute lung injury regions, releasing cordycepin to exert anti‐inflammatory effects.^[^ ^64^ ^]^ Ac‐PGP peptide‐modified tetrahedral framework nucleic acid NPs (APTB) are taken up and encapsulated by neutrophils, which are chemoattracted to inflammatory sites to release baicalein, promoting macrophage polarization from pro‐inflammatory M1 to anti‐inflammatory M2 phenotypes.^[^ ^45^ ^]^ Sun et al. created a novel drug delivery system (RBC@SIM‐PEI‐PPNPs) that transports simvastatin to pneumonia regions for anti‐inflammatory efficacy.^[^ ^35^ ^]^ Among diverse strategies for nanomaterial‐based pneumonia treatment, targeted hormone delivery represents another vital approach.

Based on the important role of hormone therapy in the treatment of pneumonia,^[^ ^187^ ^]^ NPs enable precise glucocorticoid delivery to pneumonia sites for anti‐inflammatory action. Drug‐loaded liposomal NPs (n‐DOCPs) are internalized by activated neutrophils and release encapsulated dexamethasone via NETs in pneumonia regions.^[^ ^63^ ^]^ Ding et al. designed MPSS‐loaded chitosan NPs (MPSS‐CSNPs) that leverage red blood cell‐mediated transport to enhance glucocorticoid accumulation in lungs, reducing hormone dependency and severe side effects caused by high‐dose glucocorticoid use in COVID‐19 pneumonia treatment.^[^ ^33^ ^]^

In summary, BCM‐NHS exhibits multifaceted advantages and potential in pneumonia therapy, offering vast prospects for future medical research and clinical applications.

## 6 Discussion

The application of nanomaterials in drug delivery has opened new avenues for precision medicine while also presenting unique challenges. Meticulously designed NPs can overcome the limitations of traditional drug delivery systems in addressing patient heterogeneity and disease complexity. By modulating NP characteristics such as size, shape, charge, surface properties, and responsiveness, their delivery efficacy can be optimized for specific therapeutic applications and patient populations.^[^ ^140^, ^170^, ^188^, ^189^ ^]^ However, conventional nanodrugs face significant challenges, including short circulation time, inadequate targeting specificity, and off‐target effects, which hinder their clinical translation. To address these limitations, researchers have developed BCM‐NHS. These systems leverage circulating cells, proteins, or bacteria as natural “mobile carriers” to enhance drug delivery. The BCM‐NHS approach offers several key advantages: 1) Immune evasion and prolonged circulation; 2) Dynamic targeting; 3) Biocompatibility/biodegradability; 4) Naturally optimized biological interfaces. Together, these advantages make BCM‐NHS a highly promising strategy for achieving precision‐targeted therapies.^[^ ^11^, ^190^, ^191^ ^]^

Building upon this research background, this article provides a systematic review and comprehensive elucidation of BCM‐NHS in drug delivery systems, covering its methodologies, mechanisms, and therapeutic applications. First, the approach utilizes two distinct techniques: the surface‐based “Backpack” method, which relies on ligand‐receptor binding, covalent conjugation, and non‐covalent binding, and the encapsulated “Trojan horse” approach, which employs neutrophils and monocytes/macrophages as natural delivery vehicles. Second, the system leverages diverse biological carriers, both cellular carriers (e.g., red blood cells, leukocytes) and non‐cellular carriers (e.g., albumin, bacteria). Third, the physical properties of NPs—including surface charge, size, shape, rigidity, and surface ligands—determine delivery efficiency. The release mechanisms are responsive to intracellular stimuli (e.g., pH variations, redox reactions, enzymatic activity, thermal changes, and neutrophil extracellular traps) or external stimuli, enabling spatiotemporal control. Finally, this strategy demonstrates significant therapeutic potential, particularly in treating tumor, ischemic stroke, and pneumonia. Compared to conventional methods, the BCM‐NHS offers superior pharmacokinetics, reduced systemic toxicity, and enhanced targeting precision.

Despite remarkable successes, clinical translation of BCM‐NHS faces multiple challenges: 1) Safety and efficacy validation: we need to ensure their safety and efficacy in clinical applications, rigorous evaluations are required, including biocompatibility studies, immunogenicity assessments, long‐term safety and toxicity evaluations, in vivo biodistribution and clearance pathways. 2) Functional optimization: it is important to enhance nanomaterial targeting efficiency and specificity toward circulating cells/proteins, achieving precise controlled drug release and optimizing non‐invasive administration routes. 3) Translational barriers and scalability barriers: Current research primarily relies on murine models, which cannot fully replicate the complexity and heterogeneity of human diseases. Concurrently, the absence of standardized scale‐up protocols from bench‐scale development to good manufacturing practice (GMP)‐certified manufacturing continues to hinder clinical translation.

Nanomaterials present significant challenges in designing nanoparticles with specific properties due to their vast combinatorial space and complex structure‐function relationships. The integration of machine learning and artificial intelligence (AI) to optimize nanocarrier design has become a research hotspot for achieving precise drug delivery.^[^ ^192^, ^193^, ^194^, ^195^, ^196^, ^197^ ^]^ Future applications of AI in nanomaterials include: 1) Material screening and property prediction: AI analyzes material databases to predict drug‐carrier binding energy for optimizing drug loading efficiency, while also predicting the relationship between physicochemical properties (e.g., particle size, surface charge, degradation rate) of nanocarrier materials (such as liposomes, polymers, and metal nanoparticles) and their biocompatibility. 2) Targeting optimization: By combining single‐cell RNA sequencing data and patient imaging, AI can identify disease‐specific biomarkers (e.g., tumor surface receptors) to guide the design of nanocarrier surface ligands (e.g., antibodies, peptides), and optimize chemotaxis and targeting (e.g., magnetic navigation, chemokine gradient response) through molecular dynamics simulations of nanocarrier movement in vascular networks. 3) Structure‐function relationship modeling: AI establishes mapping relationships between nanocarrier structures (e.g., porosity, shape, surface modifications) and drug release kinetics (e.g., pH‐responsive, enzyme‐triggered). 4) Patient‐specific optimization: Virtual models based on patient genomic and pathological data enable AI to simulate pharmacokinetics (e.g., hepatic clearance) of different nanocarriers in individuals, recommending personalized dosages and carrier types. 5) Implantable sensor feedback: Real‐time monitoring of lesion microenvironment parameters (e.g., pH, ROS) by implantable sensors allows AI algorithms to dynamically adjust nanocarrier drug release rates, forming adaptive therapeutic closed‐loop systems. 6) Manufacturing process optimization: AI can automatically generate microfluidic channel structures to improve nanocarrier synthesis uniformity, while computer vision monitors nanoparticle aggregation during synthesis for real‐time parameter adjustment. Current challenges include data scarcity, biological complexity, and ethical/safety concerns. Overall, the deep integration of AI with nanotechnology is transforming drug delivery systems from “empirical trial‐and‐error” to a “computationally‐driven” paradigm, though interdisciplinary collaboration (among biologists, algorithm engineers, and clinicians) remains crucial to overcome translational bottlenecks.

Besides AI applications, future research breakthroughs should focus on two additional critical dimensions: 1) Enhanced evaluation systems: The adoption of clinically relevant animal models will provide deeper insights into immune‐related adverse effects and therapeutic efficacy. Furthermore, establishing a robust safety assessment framework that evaluates acute/chronic toxicity, carcinogenic potential, and immunomodulatory effects is paramount. 2) Standardized manufacturing: The clinical translation of BCM‐NHS requires the development of standardized production processes. Key priorities include minimizing batch‐to‐batch variability through rigorous quality control measures to ensure consistent product quality.

As nanotechnology continues to evolve alongside our deepening understanding of biological systems, the insights presented here will play a pivotal role in shaping future studies, guiding regulatory decisions, and ultimately propelling these innovations into clinical practice. With sustained interdisciplinary collaboration, BCM‐NHS are poised to overcome current limitations, revolutionizing therapeutic delivery and making transformative contributions to global healthcare.

## Conflict of Interest

The authors declare no conflict of interest.

## Supporting information



Supporting Information
