# Supplementary material for: Precision‐Guided Stealth Missiles in Biomedicine: Biological Carrier‐Mediated Nanomedicine Hitchhiking Strategy
Source: Adv Sci (Weinh). 2025 May 8;12(21):2504672. doi: 10.1002/advs.202504672 (PMC12140387; doi:10.1002/advs.202504672)
Supplement: Supplementary file 1 — Supporting Information [file ADVS-12-2504672-s001.docx]

| Table S1. The advantages and disadvantages of different biological carriers. | | | | |  |  |
| --- | --- | --- | --- | --- | --- | --- |
| Cell Type | **Size (μm)** | **Circulation Time (days)** | **Characteristics** | **Advantages** | **Limitations** | **Reference** |
| Red Blood Cells | 7-8 | 120 | Nucleus-free; Biconcave disc shape; Simple structure; Abundant in number; Long lifespan; Unique elasticity and deformability; High biocompatibility | Immune evasion; Abundant source; Long circulation | Lack targeting specificity; Blood type compatibility | ^[1-11]^ |
| Monocytes/Macrophages | 15-20 | 3-5 | Rapid response to inflammation and infection; Low immunogenicity; Ability to cross biological barriers; Inflammation and tumor targeting capability | Immune evasion; Inflammation/tumor targeting; Endothelial penetration | Limited extraction yield; Potential immune overresponse | ^[12-19]^ |
| Neutrophils | 10-15 | 4-6 | Rapid response to inflammation and infection; Low immunogenicity; Ability to cross biological barriers; Inflammation and tumor targeting capability | Immune evasion; Inflammation/tumor targeting; Endothelial penetration | Limited extraction yield; Potential immune overresponse | ^[20-37]^ |
| Platelets | 2-3 | 8-9 | Small size; High reactivity; Relatively low rigidity of the platelet membrane; Vascular injury homing; Ability to interact with tumor cells | Immune evasion; Inflammation/tumor targeting; Vascular injury homing | Thrombosis risk | ^[13, 38-46]^ |
| Albumin | 3.5-4 | 19 | Abundant; Biocompatible; Biodegradable; Rich binding sites; Longer half-life; Minimal toxicity and immunogenicity; Permeation through leaky vessels into tumors; Accumulation in rapidly growing tumors | Immune evasion; Prolonged nanoparticle half-life; Ease of separation and cost-effectiveness; Targeting of tumor lesions | - | ^[47-49]^ |

| Table S2. Nanoparticles “hitchhike” on neutrophils for transportation. | | | | | | | | | | |
| --- | --- | --- | --- | --- | --- | --- | --- | --- | --- | --- |
| Hitchhiking pathway | **No.** | **Nanomedicine name** | **Nanoscale structure** | **Hitchhiking cells** | **Ligands on nanomedicine** | **Receptors on hitchhiking cells** | **Loaded drug name** | **Function of loaded drug** | **Application disease** | **Reference** |
| Ligand-receptor interaction | 1 | APT | Tetrahedral framework nucleic acid nanoparticles modified with ac-pgp peptide | Neutrophils | Ac-PGP peptide sequence | CXCR2 receptor | All-trans retinoic acid (ATRA) | Induce differentiation of promyelocytes into mature granulocytes, inhibit the proportion and absolute number of neutrophils | Sepsis | ^[50]^ |
|  | 2 | HPNFcN | Semiconductor polymer nanoparticles modified with SA | Neutrophils | Sialic acid (SA) | None | Ferrocene prodrug and immunotherapy drug NLG919 | Induce ferroptosis, trigger immunogenic cell death, enhance antitumor immune response | Glioblastoma | ^[51]^ |
|  | 3 | T-TMP | PLGA nanoparticles modified with CFLFLF peptide | Neutrophils | CFLFLF peptide | FPR receptor | Ligustrazine | Reduce reperfusion injury | Ischemic stroke | ^[52]^ |
|  | 4 | h-ANEs | Selenium (Se)-containing nano-integrated cascade enzymes (h-ANEs) hybridized with antioxidant enzymes catalase (CAT), superoxide dismutase 1 (SOD1), and bovine serum albumin (BSA) | Neutrophils | - | Fcγ receptor | Catalase (CAT), superoxide dismutase (SOD1), selenium (Se) | Antioxidation and anti-ferroptosis, reduce reperfusion injury | Ischemic stroke | ^[53]^ |
|  | 5 | NPN | Bacterial outer membrane vesicles (OMV) encapsulating photothermal transducers (PBT) | Neutrophils | Ligand-receptor | Pathogen-associated molecular patterns (PAMPs) | Cisplatin | Enhance anticancer effects through photothermal therapy combined with chemotherapy | Tumor | ^[54]^ |
|  | 6 | PM@Pic/PtCD@NP | Platinum-doped carbon dot nanozymes encapsulated by platelet membrane shell | Neutrophils | - | - | Resveratrol and platinum-doped carbon dot nanozymes (PtCD) | Clear ROS, inhibit neutrophil aggregation, alleviate ulcerative colitis | Ulcerative colitis | ^[55]^ |
|  | 7 | OMV@PGZ | Bacterial outer membrane vesicles (OMV) encapsulating pioglitazone (PGZ) | Neutrophils | Pathogen-associated molecular patterns (PAMPs) | Pattern recognition receptors (PRRs) | Pioglitazone (PGZ) | Inhibit NLRP3 inflammasome activation and ferroptosis, reduce reperfusion injury, exert neuroprotective effects | Ischemic stroke | ^[56]^ |
| Ligand-receptor mediated endocytosis and encapsulation | 8 | NPs@NEs | PLGA nanoparticles modified with cRGD Peptide | Neutrophils | cRGD peptide | L-selectin | Cabazitaxel, dexamethasone, teriparatide | Antitumor or anti-osteoporosis | Bone metastatic cancer or osteoporosis | ^[57]^ |
|  | 9 | cRGD-modified dexamethasone liposomes | Liposomal nanoparticles modified with cRGD peptide | Neutrophils | cRGD peptide | L-selectin | Dexamethasone | Anti-inflammatory effect, immune response regulation | Sepsis | ^[58]^ |
|  | 10 | cRGD-SVT-Lipo | Liposomal nanoparticles modified with cRGD peptide | Neutrophils | cRGD peptide | Integrin ανβ3 | - | Inhibit neutrophil elastase activity, reduce plaque inflammation and destruction | Atherosclerosis | ^[59]^ |
|  | 11 | c(RGDfk)-functionalized liposomes | Liposomal nanoparticles modified with cRGD peptide | Neutrophils | cRGD peptide | Integrin | Carfilzomib and BMS-202 | Immune regulation, enhanced antitumor immune response | Multiple myeloma | ^[60]^ |
|  | 12 | SPPS | Nanoparticles modified with sialic acid (SA) | Neutrophils | Sialic acid (SA) | L-selectin | siBcl-2 gene drug, polydopamine (PDA) | Block NETosis, increase bacterial survival in tumors | Tumor | ^[61]^ |
|  | 13 | D@HPB@SPM NPs | Hollow prussian blue nanoparticles core with DNase I loaded and sialic acid (SA)-modified platelet membrane shell | Neutrophils | Sialic acid (SA) | αMβ2 integrin | Deoxyribonuclease I (DNase I) | Clear ROS, degrade NETs | Ischemic stroke | ^[62]^ |
|  | 14 | n-DOCPs | Drug-loaded liposomal nanoparticles | Neutrophils | Complement fragment iC3b (attached during circulation) | CR3 receptor | Dexamethasone (Dex), antibiotics (e.g., ampicillin) | Bacterial killing | Acute lung injury | ^[63]^ |
|  | 15 | CPPC | Polydopamine (PDA) nanoparticles with anti-CD11b antibody | Neutrophils | Anti-CD11b antibody | CD11b antigen | Antibiotics, cordycepin (Cor) | Anti-inflammatory effect | Acute lung injury | ^[64]^ |
|  | 16 | Anti-Ly6G antibody-modified liposomes | Liposomes modified with anti-Ly6G antibody | Neutrophils | Anti-Ly6G antibody | Ly6G | Anticancer drug doxorubicin (DOX) and non-nucleoside STING agonist SR-717 | Inhibit tumor recurrence and metastasis | Tumor | ^[65]^ |
|  | 17 | APTB | Tetrahedral framework nucleic acid nanoparticles modified with Ac-PGP peptide | Neutrophils | Ac-PGP peptide sequence | CXCR2 receptor | Baicalin | Inhibit neutrophil inflammatory response, promote macrophage polarization | Inflammatory diseases | ^[50]^ |
|  | 22 | BSA-GOx-NPs | Bovine serum albumin nanoparticles loaded with glucose oxidase (GOx) | Neutrophils | Fcγ receptor on neutrophil surface | Fcγ receptor on neutrophils | glucose oxidase (GOx) | Catalyze glucose to produce gluconic acid and hydrogen peroxide, induce cell apoptosis by depleting glucose | Endometriosis | ^[66]^ |
|  | 24 | B-Lipo/1-MT&Cur | Bacterial membrane from attenuated salmonella VNP20009 incorporated into liposome phospholipid layer to prepare bacterial membrane-doped liposomes | Neutrophils | Pathogen-associated molecular patterns (PAMPs) | Pattern recognition receptors (PRRs) | 1-Methyl-D-tryptophan (1-MT) and curcumin (Cur) | 1-Methyl-D-tryptophan (1-MT) inhibits tryptophan metabolism pathway, curcumin (Cur) promotes immunogenic cell death | Glioblastoma | ^[67]^ |

| Table S3. Nanoparticles “hitchhike” on monocytes/macrophages for transportation. | | | | | | | | | | |
| --- | --- | --- | --- | --- | --- | --- | --- | --- | --- | --- |
| Hitchhiking pathway | **No.** | **Nanomedicine name** | **Nanoscale structure** | **Hitchhiking cells** | **Ligands on nanomedicine** | **Receptors on hitchhiking cells** | **Loaded drug name** | **Function of loaded drug** | **Application disease** | **Reference** |
| Ligand-receptor | 1 | D@MLL | Nanomaterials modified with lipid MMP-2 responsive peptide and lipoteichoic acid (LTA) | Monocytes | Lipoteichoic acid (LTA) | CD14 receptor | Doxorubicin (DOX·HCl) | Induce immunogenic cell death, promote antitumor immune response | Glioblastoma | ^[68]^ |
|  | 2 | MCP1-Gd Micelles | Micelles modified with MCP1 | Monocytes | Peptide with CCR2 binding motif (MCP1) | CCR2 receptor | Gadolinium (Gd) | Enhance MRI signal, detect metastatic LN | Metastatic lymph nodes | ^[69]^ |
|  | 3 | NPYs | Nanoparticles using yeast cell wall as a carrier | Intestinal macrophages | β-glucan in fungal cell wall | Dectin receptor on macrophages | Emodin (EMO) and asiatic acid (AA) | Modulate TGF-β/Smad signaling pathway, improve renal fibrosis | Renal fibrosis | ^[70]^ |
| Ligand-receptor mediated endocytosis and encapsulation | 4 | Liposomal nanoparticles modified with cRGD peptide | Liposomal nanoparticles modified with cRGD peptide | Monocytes/Macrophages | cRGD peptide | Integrin ανβ3 | - | Tumor targeting | Tumor | ^[60]^ |
|  | 5 | DA-βGlus/ODs/PTX | Nanoparticles modified with deaminated glucosaminoglycan (DA-βGlus) | Monocytes/Macrophages | Deaminated glucosaminoglycan (DA-βGlus) | Dectin-1 receptor | Paclitaxel (PTX) | Reduce tumor stromal fibrosis, precisely deliver Paclitaxel (PTX), inhibit tumor growth | Pancreatic cancer | ^[71]^ |
|  | 6 | 𝜷Glus-ZnD | Nanoparticles modified with β-cyclodextrin | Monocytes/Macrophages | β-cyclodextrin (βGlus) | Macrophage βGlus receptor | Chemotherapy drug doxorubicin (DOX) and zinc ions (Zn²⁺) | DOX induces tumor cell apoptosis, Zn²⁺ enhances therapeutic effect | Pancreatic cancer | ^[72]^ |
|  | 7 | bPEI-SS-PEG-T/NLS/DNA | Peptide segment with nuclear localization signal (NLS) and polymer nanoparticles crosslinked with tuftsin-modified disulfide bonds | Monocytes/Macrophages | Tuftsin | Macrophage tuftsin receptor | IL-10 plasmid DNA | Regulate macrophage metabolic reprogramming, inhibit mTOR activity, induce apoptosis | Rheumatoid arthritis | ^[73]^ |
|  | 8 | SH-β CD; SH-Fc | (1) Copper sulfide nanoparticles modified with β-cyclodextrin (SH-β CD); (2) Copper sulfide nanoparticles modified with bipyridine (Fc) (SH-Fc) | Monocytes/Macrophages | - | - | Copper sulfide (CuS) | Penetrate tumor tissue, produce photothermal effect, induce tumor cell apoptosis | Tumor | ^[74]^ |
|  | 9 | Citop-NMs | Nanomicelles based on amphiphilic polymer DSPP | Monocytes/Macrophages | - | - | Citral and sulfo-captopril | Reduce NLRP3-dependent IL-1β secretion, lower blood pressure, and have anti-atherosclerotic effects | Atherosclerosis | ^[75]^ |
|  | 10 | GNPs | Gold nanoparticles (GNPs) coated with membrane from E. coli outer membrane vesicles (OMVs) | Macrophages | Pathogen-associated molecular patterns (PAMPs) | Pattern recognition receptors (PRRs) | Gold nanoparticles | Induce tumor cell apoptosis through photothermal effect and immunogenic cell death, activate antitumor immune response | Tumor | ^[76]^ |
|  | 11 | DI/Abs | Apoptotic bodies (DI/Abs) loaded with chemotherapy drug doxorubicin (Dox) and photothermal therapy drug indocyanine green (ICG) | Macrophages | Pathogen-associated molecular patterns (PAMPs) | Pattern recognition receptors (PRRs) | Doxorubicin (Dox) and indocyanine green (ICG) | Indocyanine Green (ICG) for photothermal-chemotherapy combination treatment, release doxorubicin to produce chemotherapeutic effects on tumor cells | Glioma | ^[77]^ |
|  | 12 | Arsenic/AB biomimetic formulation | Biomimetic nanoparticles combining arsenic with apoptotic cells | Macrophages | - | - | Arsenic | Induce toxicity or apoptosis in tumor cells | Hepatocellular carcinoma | ^[78]^ |
|  | 13 | TDHP | Dual-mode biosensor based on PNA/peptide copolymer and DNA tetrahedron | Macrophages | - | - | Multisegment PNA/peptide copolymer (4PD) | Tumor imaging and urine analysis | Tumor | ^[79]^ |

| Table S4. Nanoparticles “hitchhike” on red blood cells for transportation. | | | | | | | | | | |
| --- | --- | --- | --- | --- | --- | --- | --- | --- | --- | --- |
| Hitchhiking pathway | **No.** | **Nanomedicine name** | **Nanoscale structure** | **Hitchhiking cells** | **Ligands on nanomedicine** | **Receptors on hitchhiking cells** | **Loaded drug name** | **Function of loaded drug** | **Application disease** | **Reference** |
| Ligand-receptor | 1 | SHIDS | Nanoparticles modified with modified chitosan | Red blood cells | Aminoglucose on chitosan | GLUT | Insulin, glucose oxidase (GOx), catalase (CAT) | Closed-loop glucose regulation, achieve long-term automatic blood glucose control | Diabetes | ^[80]^ |
| Non-covalent interaction | 2 | OPDEA-PS | Nanomicelles based on OPDEA | Red blood cells | - | - | Clarithromycin | Antibacterial effect | Pneumonia | ^[81]^ |
|  | 3 | Dox-IONPs | Nanoparticles based on multi-particle iron oxide (MIO) | Red blood cells | - | - | Doxorubicin (Dox) | Under the action of an alternating magnetic field, multi-particle iron oxide (MIO) generates heat, thereby releasing tumor-associated antigens | Tumor pulmonary metastasis | ^[82]^ |
|  | 4 | MPSS-CSNPs | Chitosan nanoparticles | Red blood cells | - | - | Methylprednisolone succinate sodium (MPSS) | Anti-inflammatory effect of the hormone | COVID-19 pneumonia | ^[83]^ |
|  | 5 | G-OVA-PLGA | β-glucan-ovalbumin (OVA) complex nanoparticles encapsulated by PLGA | Red blood cells | - | - | β-glucan and ovalbumin (OVA) | (1) β-glucan promotes macrophage activation towards the M1 type; (2) Ovalbumin (OVA) as a model antigen can be taken up and processed by antigen-presenting cells to activate T cells | COVID-19 pneumonia | ^[84]^ |
|  | 6 | SIM-PEI-PPNPs | pH-responsive cationic simvastatin nanoparticles | Red blood cells | - | - | Simvastatin | Alleviate acute respiratory distress syndrome | Acute respiratory distress syndrome (ARDS) | ^[85]^ |
|  | 7 | IVM-PNPs; IVM-CSPNPs | Ivermectin nanoparticles encapsulated by PLGA or chitosan | Red blood cells | - | - | Ivermectin | Antiviral and anti-inflammatory effects | COVID-19 pneumonia | ^[86]^ |
|  | 8 | β-cyclodextrin-modified ferene liposomes | Liposomes loaded with curcumin modified by ferene (Fc) | Red blood cells | - | - | Curcumin | Anti-inflammatory effect | Acute pneumonia | ^[87]^ |
| Chemical bonding | 9 | SS31-Rapa | Conjugate nanoparticles (SS31-Rapa) formed by a cleavable linker between SS31 (mitochondria-targeted antioxidant tetrapeptide) and rapamycin (autophagy inducer) | Red blood cells | - | - | SS31 (mitochondria-targeted antioxidant tetrapeptide) and rapamycin (autophagy inducer) | Induce autophagy | Acute kidney injury (AKI) | ^[88]^ |

| Table S5. Nanoparticles “hitchhike” on albumin for transportation. | | | | | | | | | |
| --- | --- | --- | --- | --- | --- | --- | --- | --- | --- |
| Hitchhiking pathway | No. | Nanomedicine name | Nanosccture | Hitchhiking cells | Key role of nanomedicine | Loaded drug name | Function of loaded drug | Application disease | Reference |
| Covalent bonding | 1 | IR1080 | H-aggregate and albumin covalently bonded | Albumin | - | Near-infrared window II (NIR-II) fluorescent probe IR1080 | Improve detection rate and edge delineation ability, enhance contrast between tumor and normal tissue | Tumor micrometastasis | ^[89]^ |
|  | 2 | Nutri-hijacker | Albumin covalently modified by biguanide drugs and flavonoids | Albumin | - | Biguanide drugs, Flavonoids | Biguanide drugs inhibit glycolysis, flavonoids inhibit glutaminolysis | Pancreatic ductal adenocarcinoma | ^[90]^ |
|  | 3 | EB-ss-DM1 | Evans blue (EB) and maytansine (DM1) connected through a responsive disulfide bond, EB covalently bonded with albumin | Albumin | - | Evans blue (EB) and maytansine (DM1) | Cytotoxic effect, induce tumor cell death | Tumor | ^[91]^ |
| Non-covalent interaction | 4 | PC, PY | Paclitaxel prodrug conjugated with fatty acid chains | Albumin | - | Paclitaxel | Antitumor | Tumor | ^[92]^ |
|  | 5 | rTCS-PTN-ABD | Fusion protein integrating albumin-binding domain (ABD), legumain (a cysteine protease), and trichosanthin (TCS) | Albumin | Albumin-binding domain (ABD) | Trichosanthin (TCS) | Inhibit tumor cell protein synthesis | Tumor | ^[93]^ |

| Table S6. Nanoparticles “hitchhike” on bacteria for transportation. | | | | | | | |
| --- | --- | --- | --- | --- | --- | --- | --- |
| No. | **Nanomedicine name** | **Nanoscale structure** | **Hitchhiking cells** | **Loaded drug name** | **Function of loaded drug** | **Application disease** | **English title of Reference** |
| 1 | AZCE-MN | Gold@Cerium-Zinc Composite Core-Shell Nanoparticles (Au@Zn/CeO) combined with engineered E. coli AZCE | E. coli | Zn²⁺, Ce³⁺/Ce⁴⁺ | Induce mitochondrial dysfunction, enhance ROS generation, photothermal therapy | Triple-negative breast cancer | ^[94]^ |
| 2 | P-HfO_2_ Nanoparticles (NPs) | P-HfO_2_ Nanoparticles (NPs) | Probiotics | Hafnium (Hf) | For gastrointestinal imaging | For gastrointestinal imaging | ^[95]^ |
| 3 | [PSB@Nb1.33C](mailto:PSB@Nb1.33C)/mRNA | Nanoparticles composed of photosynthetic bacteria (PSB), a new type of two-dimensional material (Nb1.33C), and tumor-associated antigen mRNA (WT1 mRNA) | Photosynthetic bacteria (PSB) | New type of two-dimensional material (Nb1.33C) and tumor-associated antigen mRNA (WT1 mRNA) | Encode tumor-associated antigens, activate immune response | Tumor | ^[96]^ |

[1] LUTZ H U, BOGDANOVA A. Mechanisms tagging senescent red blood cells for clearance in healthy humans [J]. Front Physiol, 2013, 4: 387. http://doi.org/10.3389/fphys.2013.00387.

[2] SOSALE N G, ROUHIPARKOUHI T, BRADSHAW A M, et al. Cell rigidity and shape override CD47's "self"-signaling in phagocytosis by hyperactivating myosin-II [J]. Blood, 2015, 125(3): 542-52. http://doi.org/10.1182/blood-2014-06-585299.

[3] BANERJEE R, KHANDELWAL S, KOZAKAI Y, et al. CD47 regulates the phagocytic clearance and replication of the Plasmodium yoelii malaria parasite [J]. Proc Natl Acad Sci U S A, 2015, 112(10): 3062-7. http://doi.org/10.1073/pnas.1418144112.

[4] MISRA R, SANJANA SHARATH N. Red blood cells based nanotheranostics: A smart biomimetic approach for fighting against cancer [J]. Int J Pharm, 2024, 661: 124401. http://doi.org/10.1016/j.ijpharm.2024.124401.

[5] KHANDELWAL S, VAN ROOIJEN N, SAXENA R K. Reduced expression of CD47 during murine red blood cell (RBC) senescence and its role in RBC clearance from the circulation [J]. Transfusion, 2007, 47(9): 1725-32. http://doi.org/10.1111/j.1537-2995.2007.01348.x.

[6] TSAI R K, RODRIGUEZ P L, DISCHER D E. Self inhibition of phagocytosis: the affinity of 'marker of self' CD47 for SIRPalpha dictates potency of inhibition but only at low expression levels [J]. Blood Cells Mol Dis, 2010, 45(1): 67-74. http://doi.org/10.1016/j.bcmd.2010.02.016.

[7] XIA Q, ZHANG Y, LI Z, et al. Red blood cell membrane-camouflaged nanoparticles: a novel drug delivery system for antitumor application [J]. Acta Pharm Sin B, 2019, 9(4): 675-89. http://doi.org/10.1016/j.apsb.2019.01.011.

[8] MOHANDAS N, GALLAGHER P G. Red cell membrane: past, present, and future [J]. Blood, 2008, 112(10): 3939-48. http://doi.org/10.1182/blood-2008-07-161166.

[9] BOSMAN G J, WILLEKENS F L, WERRE J M. Erythrocyte aging: a more than superficial resemblance to apoptosis? [J]. Cell Physiol Biochem, 2005, 16(1-3): 1-8. http://doi.org/10.1159/000087725.

[10] BRENNER J S, MITRAGOTRI S, MUZYKANTOV V R. Red Blood Cell Hitchhiking: A Novel Approach for Vascular Delivery of Nanocarriers [J]. Annu Rev Biomed Eng, 2021, 23: 225-48. http://doi.org/10.1146/annurev-bioeng-121219-024239.

[11] DENG J, XU S, HU W, et al. Tumor targeted, stealthy and degradable bismuth nanoparticles for enhanced X-ray radiation therapy of breast cancer [J]. Biomaterials, 2018, 154: 24-33. http://doi.org/10.1016/j.biomaterials.2017.10.048.

[12] MURRAY P J, ALLEN J E, BISWAS S K, et al. Macrophage activation and polarization: nomenclature and experimental guidelines [J]. Immunity, 2014, 41(1): 14-20. http://doi.org/10.1016/j.immuni.2014.06.008.

[13] WANG H, LIU Y, HE R, et al. Cell membrane biomimetic nanoparticles for inflammation and cancer targeting in drug delivery [J]. Biomater Sci, 2020, 8(2): 552-68. http://doi.org/10.1039/c9bm01392j.

[14] JIN K, LUO Z, ZHANG B, et al. Biomimetic nanoparticles for inflammation targeting [J]. Acta Pharm Sin B, 2018, 8(1): 23-33. http://doi.org/10.1016/j.apsb.2017.12.002.

[15] COOMBS C, GEORGANTZOGLOU A, WALKER H A, et al. Chemokine receptor trafficking coordinates neutrophil clustering and dispersal at wounds in zebrafish [J]. Nat Commun, 2019, 10: 17. http://doi.org/10.1038/s41467-019-13107-3.

[16] BARLIC J, MURPHY P M. An oxidized lipid-peroxisome proliferator-activated receptor γ-chemokine pathway in the regulation of macrophage-vascular smooth muscle cell adhesion [J]. Trends Cardiovasc Med, 2007, 17(8): 269-74. http://doi.org/10.1016/j.tcm.2007.09.004.

[17] DIB K, TIKHONOVA I G, IVETIC A, et al. The cytoplasmic tail of L-selectin interacts with the adaptor-protein complex AP-1 subunit μ1A via a novel basic binding motif [J]. J Biol Chem, 2017, 292(16): 6703-14. http://doi.org/10.1074/jbc.M116.768598.

[18] PHAM K, HUYNH D, LE L, et al. E-cigarette promotes breast carcinoma progression and lung metastasis: Macrophage-tumor cells crosstalk and the role of CCL5 and VCAM-1 [J]. Cancer Lett, 2020, 491: 132-45. http://doi.org/10.1016/j.canlet.2020.08.010.

[19] CAO H, DAN Z, HE X, et al. Liposomes Coated with Isolated Macrophage Membrane Can Target Lung Metastasis of Breast Cancer [J]. ACS Nano, 2016, 10(8): 7738-48. http://doi.org/10.1021/acsnano.6b03148.

[20] KOLACZKOWSKA E, KUBES P. Neutrophil recruitment and function in health and inflammation [J]. Nat Rev Immunol, 2013, 13(3): 159-75. http://doi.org/10.1038/nri3399.

[21] SILVESTRE-ROIG C, FRIDLENDER Z G, GLOGAUER M, et al. Neutrophil Diversity in Health and Disease [J]. Trends Immunol, 2019, 40(7): 565-83. http://doi.org/10.1016/j.it.2019.04.012.

[22] WANG M H, JIN Z H, HUANG H Y, et al. Neutrophil hitchhiking: Riding the drug delivery wave to treat diseases [J]. Drug Dev Res, 2024, 85(2): 17. http://doi.org/10.1002/ddr.22169.

[23] PILLAY J, DEN BRABER I, VRISEKOOP N, et al. In vivo labeling with 2H2O reveals a human neutrophil lifespan of 5.4 days [J]. Blood, 2010, 116(4): 625-7. http://doi.org/10.1182/blood-2010-01-259028.

[24] WU J H, MA T, ZHU M N, et al. Nanotechnology reinforced neutrophil-based therapeutic strategies for inflammatory diseases therapy [J]. Nano Today, 2022, 46: 22. http://doi.org/10.1016/j.nantod.2022.101577.

[25] SHEN J R, ZHOU Y, YIN L C. Nano/genetically engineered cells for immunotherapy [J]. BMEMat, 2024: 37. http://doi.org/10.1002/bmm2.12112.

[26] LIU Y, SONG R, ZHAO L, et al. m(6)A demethylase ALKBH5 is required for antibacterial innate defense by intrinsic motivation of neutrophil migration [J]. Signal Transduct Target Ther, 2022, 7(1): 194. http://doi.org/10.1038/s41392-022-01020-z.

[27] XIE Y, ZHOU T, LI X, et al. Targeting ESE3/EHF With Nifurtimox Inhibits CXCR2(+) Neutrophil Infiltration and Overcomes Pancreatic Cancer Resistance to Chemotherapy and Immunotherapy [J]. Gastroenterology, 2024, 167(2): 281-97. http://doi.org/10.1053/j.gastro.2024.02.046.

[28] GIRBL T, LENN T, PEREZ L, et al. Distinct Compartmentalization of the Chemokines CXCL1 and CXCL2 and the Atypical Receptor ACKR1 Determine Discrete Stages of Neutrophil Diapedesis [J]. Immunity, 2018, 49(6): 1062-76.e6. http://doi.org/10.1016/j.immuni.2018.09.018.

[29] DEL PRETE A, MARTíNEZ-MUñOZ L, MAZZON C, et al. The atypical receptor CCRL2 is required for CXCR2-dependent neutrophil recruitment and tissue damage [J]. Blood, 2017, 130(10): 1223-34. http://doi.org/10.1182/blood-2017-04-777680.

[30] CAPPENBERG A, MARGRAF A, THOMAS K, et al. L-selectin shedding affects bacterial clearance in the lung: a new regulatory pathway for integrin outside-in signaling [J]. Blood, 2019, 134(17): 1445-57. http://doi.org/10.1182/blood.2019000685.

[31] LIU Z, YAGO T, ZHANG N, et al. L-selectin mechanochemistry restricts neutrophil priming in vivo [J]. Nat Commun, 2017, 8: 15196. http://doi.org/10.1038/ncomms15196.

[32] STADTMANN A, GERMENA G, BLOCK H, et al. The PSGL-1-L-selectin signaling complex regulates neutrophil adhesion under flow [J]. J Exp Med, 2013, 210(11): 2171-80. http://doi.org/10.1084/jem.20130664.

[33] MCEVER R P. Selectins: initiators of leucocyte adhesion and signalling at the vascular wall [J]. Cardiovasc Res, 2015, 107(3): 331-9. http://doi.org/10.1093/cvr/cvv154.

[34] WEN L, MOSER M, LEY K. Molecular mechanisms of leukocyte β2 integrin activation [J]. Blood, 2022, 139(24): 3480-92. http://doi.org/10.1182/blood.2021013500.

[35] MITROULIS I, ALEXAKI V I, KOURTZELIS I, et al. Leukocyte integrins: role in leukocyte recruitment and as therapeutic targets in inflammatory disease [J]. Pharmacol Ther, 2015, 147: 123-35. http://doi.org/10.1016/j.pharmthera.2014.11.008.

[36] LI X, QIAO Q, LIU X, et al. Engineered Biomimetic Nanovesicles Based on Neutrophils for Hierarchical Targeting Therapy of Acute Respiratory Distress Syndrome [J]. ACS Nano, 2024, 18(2): 1658-77. http://doi.org/10.1021/acsnano.3c09848.

[37] ZHANG Q, HU C, FENG J, et al. Anti-inflammatory mechanisms of neutrophil membrane-coated nanoparticles without drug loading [J]. J Control Release, 2024, 369: 12-24. http://doi.org/10.1016/j.jconrel.2024.03.030.

[38] BOILARD E, NIGROVIC P A, LARABEE K, et al. Platelets amplify inflammation in arthritis via collagen-dependent microparticle production [J]. Science, 2010, 327(5965): 580-3. http://doi.org/10.1126/science.1181928.

[39] MACHLUS K R, ITALIANO J E, JR. The incredible journey: From megakaryocyte development to platelet formation [J]. J Cell Biol, 2013, 201(6): 785-96. http://doi.org/10.1083/jcb.201304054.

[40] QI C, LI B, GUO S, et al. P-Selectin-Mediated Adhesion between Platelets and Tumor Cells Promotes Intestinal Tumorigenesis in Apc(Min/+) Mice [J]. Int J Biol Sci, 2015, 11(6): 679-87. http://doi.org/10.7150/ijbs.11589.

[41] WALLIS S, WOLSKA N, ENGLERT H, et al. A peptide from the staphylococcal protein Efb binds P-selectin and inhibits the interaction of platelets with leukocytes [J]. J Thromb Haemost, 2022, 20(3): 729-41. http://doi.org/10.1111/jth.15613.

[42] BORSIG L, WONG R, FERAMISCO J, et al. Heparin and cancer revisited: mechanistic connections involving platelets, P-selectin, carcinoma mucins, and tumor metastasis [J]. Proc Natl Acad Sci U S A, 2001, 98(6): 3352-7. http://doi.org/10.1073/pnas.061615598.

[43] LIANG H, YANG C X, ZHANG B, et al. Sevoflurane attenuates platelets activation of patients undergoing lung cancer surgery and suppresses platelets-induced invasion of lung cancer cells [J]. J Clin Anesth, 2016, 35: 304-12. http://doi.org/10.1016/j.jclinane.2016.08.008.

[44] TESFAMARIAM B. Involvement of platelets in tumor cell metastasis [J]. Pharmacol Ther, 2016, 157: 112-9. http://doi.org/10.1016/j.pharmthera.2015.11.005.

[45] SUN L, YU Y, PENG Y, et al. Platelet Membrane-Derived Nanodiscs for Neutralization of Endogenous Autoantibodies and Exogenous Virulence Factors [J]. Small, 2024, 20(18): e2308327. http://doi.org/10.1002/smll.202308327.

[46] KOUPENOVA M, VITSEVA O, MACKAY C R, et al. Platelet-TLR7 mediates host survival and platelet count during viral infection in the absence of platelet-dependent thrombosis [J]. Blood, 2014, 124(5): 791-802. http://doi.org/10.1182/blood-2013-11-536003.

[47] SPADA A, EMAMI J, TUSZYNSKI J A, et al. The Uniqueness of Albumin as a Carrier in Nanodrug Delivery [J]. Mol Pharm, 2021, 18(5): 1862-94. http://doi.org/10.1021/acs.molpharmaceut.1c00046.

[48] SPADA A, EMAMI J, TUSZYNSKI J A, et al. The Uniqueness of Albumin as a Carrier in Nanodrug Delivery [J]. Mol Pharm, 2021, 18(5): 1862-94. http://doi.org/10.1021/acs.molpharmaceut.1c00046.

[49] GUPTA N, HATOUM H, DY G K. First line treatment of advanced non-small-cell lung cancer - specific focus on albumin bound paclitaxel [J]. Int J Nanomedicine, 2014, 9: 209-21. http://doi.org/10.2147/ijn.S41770.

[50] ZHOU M, TANG Y L, LU Y F, et al. Framework Nucleic Acid-Based and Neutrophil-Based Nanoplatform Loading Baicalin with Targeted Drug Delivery for Anti-Inflammation Treatment [J]. ACS Nano, 2025, 19(3): 3455-69. http://doi.org/10.1021/acsnano.4c12917.

[51] ZHU A N, TU W Z, DING M B, et al. X-ray-activatable hitchhiking polymer nanodrugs enable controllable ferroptosis and immunization for orthotopic glioma rejection [J]. Chem Eng J, 2024, 497: 15. http://doi.org/10.1016/j.cej.2024.154652.

[52] MU Q C, YAO K, SYEDA M Z, et al. Ligustrazine Nanoparticle Hitchhiking on Neutrophils for Enhanced Therapy of Cerebral Ischemia-Reperfusion Injury [J]. Adv Sci, 2023, 10(19): 10. http://doi.org/10.1002/advs.202301348.

[53] WANG W, ZHANG Z, LIU Y, et al. Nano-integrated cascade antioxidases opsonized by albumin bypass the blood-brain barrier for treatment of ischemia-reperfusion injury [J]. Biomater Sci, 2022, 10(24): 7103-16. http://doi.org/10.1039/d2bm01401g.

[54] LI M, LI S, ZHOU H, et al. Chemotaxis-driven delivery of nano-pathogenoids for complete eradication of tumors post-phototherapy [J]. Nat Commun, 2020, 11(1): 1126. http://doi.org/10.1038/s41467-020-14963-0.

[55] YAN X J, SONG J, ZHANG Y J, et al. Platelet-inspired nanomedicine targeting activated neutrophils to alleviate ulcerative colitis by free radicals scavenging and controlled neutrophil swarming [J]. Nano Today, 2024, 54: 17. http://doi.org/10.1016/j.nantod.2023.102139.

[56] PAN J, WANG Z, HUANG X, et al. Bacteria-Derived Outer-Membrane Vesicles Hitchhike Neutrophils to Enhance Ischemic Stroke Therapy [J]. Adv Mater, 2023, 35(38): e2301779. http://doi.org/10.1002/adma.202301779.

[57] LUO Z Y, LU Y C, SHI Y Y, et al. Neutrophil hitchhiking for drug delivery to the bone marrow [J]. Nat Nanotechnol, 2023, 18(6): 647-+. http://doi.org/10.1038/s41565-023-01374-7.

[58] MATHUR R, ELSAFY S, PRESS A T, et al. Neutrophil Hitchhiking Enhances Liposomal Dexamethasone Therapy of Sepsis [J]. ACS Nano, 2024, 18(42): 28866-80. http://doi.org/10.1021/acsnano.4c09054.

[59] SHI Y, DONG M, WU Y, et al. An elastase-inhibiting, plaque-targeting and neutrophil-hitchhiking liposome against atherosclerosis [J]. Acta Biomater, 2024, 173: 470-81. http://doi.org/10.1016/j.actbio.2023.11.020.

[60] SOFIAS A M, TONER Y C, MEERWALDT A E, et al. Tumor Targeting by α(v)β(3)-Integrin-Specific Lipid Nanoparticles Occurs via Phagocyte Hitchhiking [J]. ACS Nano, 2020, 14(7): 7832-46. http://doi.org/10.1021/acsnano.9b08693.

[61] ZHAO Y, LI M, GUO Y, et al. Neutrophil hitchhiking nanoparticles enhance bacteria-mediated cancer therapy via NETosis reprogramming [J]. J Control Release, 2024, 367: 661-75. http://doi.org/10.1016/j.jconrel.2024.01.068.

[62] SONG J Y, YANG G, SONG Y, et al. Neutrophil Hitchhiking Biomimetic Nanozymes Prime Neuroprotective Effects of Ischemic Stroke in a Tailored "Burning the Bridges" Manner [J]. Advanced Functional Materials, 2024, 34(32): 15. http://doi.org/10.1002/adfm.202315275.

[63] LI S, LI M, HUO S, et al. Voluntary-Opsonization-Enabled Precision Nanomedicines for Inflammation Treatment [J]. Adv Mater, 2021, 33(3): e2006160. http://doi.org/10.1002/adma.202006160.

[64] GAO M Q, FAN H Z, YU S F, et al. Neutrophil-mediated cordycepin-based nanoparticles for targeted treatment of acute lung injury [J]. Chem Eng J, 2025, 506: 14. http://doi.org/10.1016/j.cej.2025.159942.

[65] GAO Z L, WANG N, MA Y, et al. Targeting neutrophils potentiates hitchhiking delivery of drugs and agonists for postsurgical chemo-immunotherapy [J]. Nano Today, 2024, 54: 15. http://doi.org/10.1016/j.nantod.2023.102096.

[66] ZHU S, ZHANG J, XUE N, et al. Highly specific neutrophil-mediated delivery of albumin nanoparticles to ectopic lesion for endometriosis therapy [J]. J Nanobiotechnology, 2023, 21(1): 81. http://doi.org/10.1186/s12951-023-01831-4.

[67] LIU X P, YI X, GU J Y, et al. Immunoregulatory liposomes hitchhiking on neutrophils for enhanced carbon ion radiotherapy-assisted immunotherapy of glioblastoma [J]. Nano Today, 2023, 53: 14. http://doi.org/10.1016/j.nantod.2023.102037.

[68] KUANG J, RAO Z Y, ZHENG D W, et al. Nanoparticles Hitchhike on Monocytes for Glioblastoma Treatment after Low-Dose Radiotherapy [J]. ACS Nano, 2023, 17(14): 13333-47. http://doi.org/10.1021/acsnano.3c01428.

[69] TRAC N, CHEN Z, OH H S, et al. MRI Detection of Lymph Node Metastasis through Molecular Targeting of C-C Chemokine Receptor Type 2 and Monocyte Hitchhiking [J]. ACS Nano, 2024, 18(3): 2091-104. http://doi.org/10.1021/acsnano.3c09201.

[70] XU X, DENG G, SUN Z, et al. A Biomimetic Aggregation-Induced Emission Photosensitizer with Antigen-Presenting and Hitchhiking Function for Lipid Droplet Targeted Photodynamic Immunotherapy [J]. Adv Mater, 2021, 33(33): e2102322. http://doi.org/10.1002/adma.202102322.

[71] NGUYEN N, HOANG T M, HUANG T Y, et al. Macrophage-hitchhiked, effervescence-induced nanoemulsions for enhanced oral chemotherapy and immunotherapy: Impact on absorption route [J]. Biomaterials, 2025, 316: 123019. http://doi.org/10.1016/j.biomaterials.2024.123019.

[72] CHEN K H, NGUYEN N, HUANG T Y, et al. Macrophage-Hitchhiked Orally Administered β-Glucans-Functionalized Nanoparticles as "Precision-Guided Stealth Missiles" for Targeted Pancreatic Cancer Therapy [J]. Adv Mater, 2023, 35(40): e2304735. http://doi.org/10.1002/adma.202304735.

[73] ZHANG X T, LIU Y H, LIU W, et al. Macrophage-hitchhiking interleukin-10 plasmid DNA delivery system modulates rheumatoid arthritis microenvironment via the re-polarization of macrophages [J]. Nano Today, 2024, 54: 22. http://doi.org/10.1016/j.nantod.2023.102068.

[74] LI J, CHENG Q, YUE L, et al. Macrophage-hitchhiking supramolecular aggregates of CuS nanoparticles for enhanced tumor deposition and photothermal therapy [J]. Nanoscale Horiz, 2021, 6(11): 907-12. http://doi.org/10.1039/d1nh00291k.

[75] FU C X, TAO Y, LI Z C, et al. Circulating monocyte differentiation-activated nanoprodrugs for reprogramming macrophage immunity in atherosclerotic plaques [J]. Nano Today, 2024, 56: 13. http://doi.org/10.1016/j.nantod.2024.102304.

[76] DAPKUTE D, PLECKAITIS M, BULOTIENE D, et al. Hitchhiking Nanoparticles: Mesenchymal Stem Cell-Mediated Delivery of Theranostic Nanoparticles [J]. ACS Appl Mater Interfaces, 2021, 13(37): 43937-51. http://doi.org/10.1021/acsami.1c10445.

[77] LIU Y, HU D, GAO D, et al. Engineered apoptotic bodies hitchhiking across the blood-brain barrier achieved a combined photothermal-chemotherapeutic effect against glioma [J]. Theranostics, 2023, 13(9): 2966-78. http://doi.org/10.7150/thno.80632.

[78] LI C, ZHANG J P, YUAN Y C, et al. Macrophage-hitchhiked arsenic/AB bionic preparations for liver cancer [J]. Biomater Sci, 2023, 12(1): 187-98. http://doi.org/10.1039/d3bm01311a.

[79] WEI K, XU Y, NIE C, et al. A Multifunctional Peptide Nucleic Acid/Peptide Copolymer-Based Dual-Mode Biosensor with Macrophage-Hitchhiking for Enhanced Tumor Imaging and Urinalysis [J]. J Am Chem Soc, 2024, 146(48): 33075-83. http://doi.org/10.1021/jacs.4c10562.

[80] LI M, XU X M, SHI R Y, et al. Smart erythrocyte-hitchhiking insulin delivery system for prolonged automatic blood glucose control [J]. Biomater Sci, 2022, 10(10): 2638-49. http://doi.org/10.1039/d2bm00280a.

[81] YU H, PIAO Y, ZHANG Y, et al. Cell-Selective Binding Zwitterionic Polymeric Micelles Boost the Delivery Efficiency of Antibiotics [J]. ACS Nano, 2023, 17(22): 22430-43. http://doi.org/10.1021/acsnano.3c05181.

[82] HUYNH T M H, YALAMANDALA B N, CHIANG M R, et al. Programmed antigen capture-harnessed dendritic cells by margination-hitchhiking lung delivery [J]. J Control Release, 2023, 358: 718-28. http://doi.org/10.1016/j.jconrel.2023.05.028.

[83] DING Y, LV B, ZHENG J, et al. RBC-hitchhiking chitosan nanoparticles loading methylprednisolone for lung-targeting delivery [J]. J Control Release, 2022, 341: 702-15. http://doi.org/10.1016/j.jconrel.2021.12.018.

[84] LI S, WANG Y, LIU Q, et al. RBC-hitchhiking PLGA nanoparticles loading β-glucan as a delivery system to enhance in vitro and in vivo immune responses in mice [J]. Front Vet Sci, 2024, 11: 1462518. http://doi.org/10.3389/fvets.2024.1462518.

[85] SUN M, WEI J, SU Y, et al. Red Blood Cell-Hitchhiking Delivery of Simvastatin to Relieve Acute Respiratory Distress Syndrome [J]. Int J Nanomedicine, 2024, 19: 5317-33. http://doi.org/10.2147/ijn.S460890.

[86] XIA Y, FU S, MA Q, et al. Application of Nano-Delivery Systems in Lymph Nodes for Tumor Immunotherapy [J]. Nanomicro Lett, 2023, 15(1): 145. http://doi.org/10.1007/s40820-023-01125-2.

[87] LI J, DING Y, CHENG Q, et al. Supramolecular erythrocytes-hitchhiking drug delivery system for specific therapy of acute pneumonia [J]. J Control Release, 2022, 350: 777-86. http://doi.org/10.1016/j.jconrel.2022.08.029.

[88] YU B, LIU Y, ZHANG Y, et al. An SS31-rapamycin conjugate via RBC hitchhiking for reversing acute kidney injury [J]. Biomaterials, 2023, 303: 122383. http://doi.org/10.1016/j.biomaterials.2023.122383.

[89] XU Y, YANG C, WU Y, et al. In Situ Albumin-Hitchhiking NIR-II Probes for Accurate Detection of Micrometastases [J]. Nano Lett, 2023, 23(12): 5731-7. http://doi.org/10.1021/acs.nanolett.3c01484.

[90] HUANG Y, CHEN Y, ZHOU S, et al. Synthetically Lethal Biomimetic Nutri-hijacker Hitchhikes and Reprograms KRAS Mutation-Driven Metabolic Addictions for Pancreatic Ductal Adenocarcinoma Treatment [J]. ACS Nano, 2023, 17(14): 14014-31. http://doi.org/10.1021/acsnano.3c04069.

[91] FU S, ZHENG A, WANG L, et al. Tuneable redox-responsive albumin-hitchhiking drug delivery to tumours for cancer treatment [J]. J Mater Chem B, 2024, 12(27): 6563-9. http://doi.org/10.1039/d4tb00751d.

[92] LU S, ZHOU S, XIANG X, et al. Paclitaxel prodrug nanoparticles boost antitumor efficacy via hitchhiking of human serum albumin [J]. J Colloid Interface Sci, 2025, 679(Pt B): 144-54. http://doi.org/10.1016/j.jcis.2024.10.075.

[93] CHANG Y, YAO S, CHEN Y, et al. Genetically-engineered protein prodrug-like nanoconjugates for tumor-targeting biomimetic delivery via a SHEATH strategy [J]. Nanoscale, 2019, 11(2): 611-21. http://doi.org/10.1039/c8nr08951e.

[94] ZHANG W, JIANG Y, LIU L, et al. Implantable Microneedles Loaded with Nanoparticles Surface Engineered Escherichia coli for Efficient Eradication of Triple-Negative Breast Cancer Stem Cells [J]. Nano Lett, 2025, 25(5): 2041-51. http://doi.org/10.1021/acs.nanolett.4c06052.

[95] OSTADHOSSEIN F, MOITRA P, GUNASEELAN N, et al. Hitchhiking probiotic vectors to deliver ultra-small hafnia nanoparticles for 'Color' gastrointestinal tract photon counting X-ray imaging [J]. Nanoscale Horiz, 2022, 7(5): 533-42. http://doi.org/10.1039/d1nh00626f.

[96] ZHANG S, YU J, LIU Y, et al. Photosynthetic Bacteria-Hitchhiking 2D iMXene-mRNA Vaccine to Enable Photo-Immunogene Cancer Therapy [J]. Adv Sci (Weinh), 2024, 11(28): e2307225. http://doi.org/10.1002/advs.202307225.
